# The histone methyltransferase SUV420H2 and Heterochromatin Proteins HP1 interact but show different dynamic behaviours

**DOI:** 10.1186/1471-2121-10-41

**Published:** 2009-06-01

**Authors:** Patricia P Souza, Pamela Völkel, Dave Trinel, Julien Vandamme, Claire Rosnoblet, Laurent Héliot, Pierre-Olivier Angrand

**Affiliations:** 1 Chromatinomics, Interdisciplinary Research Institute, Université des Sciences et Technologies de Lille/CNRS USR 3078, Parc Scientifique de la Haute Borne, 50 Avenue Halley, F-59658 Villeneuve d'Ascq, France; 2 Biophotonique Cellulaire Fonctionnelle, Interdisciplinary Research Institute, Université des Sciences et Technologies de Lille/CNRS USR 3078, Parc Scientifique de la Haute Borne, 50 Avenue Halley, F-59658 Villeneuve d'Ascq, France; 3 New York University Langone Medical Center, Pathology Department, 550, First Avenue, New York, NY, 10016, USA; 4 Biotech Research & Innovation Centre, University of Copenhagen, Ole Maaløes Vej 5, DK-2200 Copenhagen, Denmark

## Abstract

**Background:**

Histone lysine methylation plays a fundamental role in chromatin organization and marks distinct chromatin regions. In particular, trimethylation at lysine 9 of histone H3 (H3K9) and at lysine 20 of histone H4 (H4K20) governed by the histone methyltransferases SUV39H1/2 and SUV420H1/2 respectively, have emerged as a hallmark of pericentric heterochromatin. Controlled chromatin organization is crucial for gene expression regulation and genome stability. Therefore, it is essential to analyze mechanisms responsible for high order chromatin packing and in particular the interplay between enzymes involved in histone modifications, such as histone methyltransferases and proteins that recognize these epigenetic marks.

**Results:**

To gain insights into the mechanisms of SUV420H2 recruitment at heterochromatin, we applied a tandem affinity purification approach coupled to mass spectrometry. We identified heterochromatin proteins HP1 as main interacting partners. The regions responsible for the binding were mapped to the heterochromatic targeting module of SUV420H2 and HP1 chromoshadow domain. We studied the dynamic properties of SUV420H2 and the HP1 in living cells using fluorescence recovery after photobleaching. Our results showed that HP1 proteins are highly mobile with different dynamics during the cell cycle, whereas SUV420H2 remains strongly bound to pericentric heterochromatin. An 88 amino-acids region of SUV420H2, the heterochromatic targeting module, recapitulates both, HP1 binding and strong association to heterochromatin.

**Conclusion:**

FRAP experiments reveal that in contrast to HP1, SUV420H2 is strongly associated to pericentric heterochromatin. Then, the fraction of SUV420H2 captured and characterized by TAP/MS is a soluble fraction which may be in a stable association with HP1. Consequently, SUV420H2 may be recruited to heterochromatin in association with HP1, and stably maintained at its heterochromatin sites in an HP1-independent fashion.

## Background

Eukaryotic DNA is packaged within the nucleus through its association with histone proteins forming the fundamental repeating unit of chromatin, the nucleosome. The nucleosome consists of 146 bp of DNA wrapped around a histone core octamer composed of two each of histones H2A, H2B, H3, and H4 [1]. Histone C- and N-terminal tails are flexible, protrude from the nucleosome octamer structure, and are subjected to post-translational modifications, including acetylation, methylation, phosphorylation, ubiquitination or sumoylation. Among these modifications, histone lysine methylation patterns have been associated with distinct chromatin states and are proposed to be major epigenetic marks that could extend the information potential of the genetic code by fixing the chromatin organization in a heritable manner (for a review [2]).

In particular, constitutive heterochromatin, considered as the part of the genome that is gene poor, transcriptionally silent and highly condensed in interphase cells, is characterized to harbour nucleosomes rich in trimethylation at lysine 9 of histone H3 (H3K9me3), trimethylation at lysine 20 of histone H4 (H4K20me3) and monomethylation at lysine 27 of histone H3 (H3K27me1) [3-5]. The histone methyltransferases SUV39H1 and SUV39H2 play a crucial role in the initial steps of heterochromatin formation in mammals by selective trimethylation of H3K9 [3,6,7]. Indeed, mice that are deficient for SUV39H activities were shown to display impaired H3K9 trimethylation at pericentric heterochromatin and were subjected to chromosomal instability [8]. The molecular mechanisms by which SUV39H1 and SUV39H2 are recruited at heterochromatin are still unknown but were suggested to be mediated by direct or indirect association with components of a RNA interference pathway [9]. According to current models, H3K9me3 marks placed by SUV39H activities stabilize heterochromatin protein 1 (HP1) binding at heterochromatin [10,11], and HP1 proteins would then recruit the histone methyltransferases SUV420H2 and SUV420H1 which in turn, trimethylate H4K20 [5,12,13]. At present, it is unclear whether SUV420H histone methyltransferases interact only temporally with chromatin to methylate H4K20 or participate in a more stable multiprotein complex together with HP1 or other chromatin proteins to support a stable heterochromatin structure.

Interestingly, maintenance of stable heterochromatin domains in living cells involves the transient binding and dynamic exchange of HP1 from chromatin [14-16] indicating that heterochromatin is not a static and inaccessible higher order conformation but is a dynamic domain of chromatin. In contrast to HP1, SUV39H1 has a significantly slower exchange rate and a substantial fraction immobile at heterochromatin [17], and nothing is known about the dynamics of the SUV420H class of histone methyltransferases.

To gain further insights into the role that SUV420H2 plays in heterochromatin, we first used a directed proteomic analysis of SUV420H2-binding proteins in cells using the tandem affinity purification (TAP)-mass spectrometry (MS) methodology [18]. We showed that HP1 proteins are the main SUV420H2 interacting partners. We then investigated the in vivo kinetics of SUV420H2 in mouse L929 cells and compared them to those of HP1 proteins. Using fluorescence recovery after photobleaching (FRAP) analysis we showed that in contrast to HP1 proteins, SUV420H2 has a slow exchange rate and is largely strongly bound at pericentric heterochromatin. Furthermore, we mapped the domain responsible for stable association to chromatin to a region of 88 amino-acids of SUV420H2, previously defined as the heterochromatic targeting module by Schotta et al. [5].

## Results and discussion

### SUV420H2 interacts with HP1 proteins

Pericentric regions, as well as telomeres, are part of the constitutive heterochromatin, which is characterized by hypermethylation of DNA, hypoacetylation and hypermethylation of histones. In particular, trimethylation of H3K9 (H3K9me3) and trimethylation of H4K20 (H4K20me3) by the SUV39H and SUV420H histone methyltransferases respectively, mark pericentric chromatin [3-5,19]. In order to gain insight into the mechanisms involved in the recruitment of SUV420H enzymes at heterochromatin and identify their interacting partners, we applied the tandem affinity purification (TAP) technology coupled to tandem mass spectrometry (MS/MS), since this approach has proven to efficiently allow the characterization of protein complexes from different cells in culture or organisms [20]. TAP/MS analysis of protein associations around SUV420H2 was performed from HeLa cells, because we have assembled a database of more than 30 TAP/MS experiments from proteins involved in chromatin regulation in this cell line. This dataset allows reliable assessment of a given interaction (data not shown).

**Figure 1:**
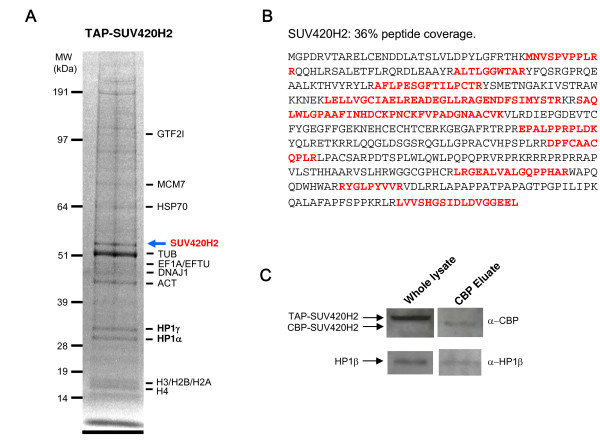
**Identification of SUV420H2 associated proteins using TAP/MS**. (**A**) TAP-SUV420H2 purification from HeLa cells. TAP-SUV420H2 protein complexes were purified from HeLa cells, separated by SDS-PAGE, and stained with colloidal Coomassie. Some of the co-purified proteins identified by LC-MS/MS are indicated. (**B**) SUV420H2 peptide coverage is 36%. The identified peptides are indicated in red bold on the SUV420H2 protein sequence. Peptides are dispersed over the full sequence of the protein. (**C**) Western blotting showing the presence of HP1β in the TAP-SUV420H2 purification. Total protein extracted from HeLa cells expressing TAP-SUV420H2 (Whole lysate; 50 μg of proteins) and TAP purified sample (CBP Eluate; 20% of the material used for MS identification) were separated by SDS-PAGE and proteins detected by Western blotting using specific antibodies. Tagged SUV420H2 was revealed using an anti-CBP antibody (α-CBP), while HP1β was revealed by an anti-HP1β antibody (α-HP1β). Note that the TEV protease-mediated cleavage of the Protein A moiety of the TAP tag during the purification procedure increases the gel mobility of the tagged SUV420H2 protein in the CBP eluate as compared to whole extract.

A retrovirus gene transfer strategy was used to generate a cell pool expressing TAP-tagged SUV420H2 fusion protein. Expanded cell pools were subjected to tandem affinity purification, a procedure consisting of two specific binding and two specific elution steps under mild conditions, which preserve the integrity of non-transient protein-protein interactions [18]. The affinity purified complexes were resolved on SDS-PAGE and Coomassie stained (Fig. 1A). Then, proteins were identified by peptide sequence determination using tandem mass spectrometry (LC-MS/MS). Most proteins were characterized by the identification of several peptides [see Additional files 1 and 2]. Details on protein identification procedures are given in the Methods section. The tagged SUV420H2 protein represents a prominent band on the gel and was identified by peptides covering 36% of its total sequence (Fig. 1B). However, SUV420H2 was not the most abundant protein recovered by TAP/MS since tubulin, which is considered as a TAP/MS contaminant, remains the most prominent identified protein. This relative low recovery of SUV420H2 by TAP is probably due to its strong association to chromatin (see below). Sonication or addition of intercalating agents such as ethidium bromide [21], prior to protein extraction, did not improve the recovery rate of SUV420H2 by TAP/MS (data not shown). In spite of a weak recovery of the bait, heterochromatin proteins (HP) 1 were identified as specific SUV420H2 interactors (Fig. 1A) by identified peptides covering 53% (HP1γ – CBX3) and 15% (HP1α – CBX5) of their total sequence, respectively. The third member of the HP1 protein family, HP1β (CBX1) was not formally identified in the purification. However, the peptide IIGATDSSGELMFLMK identified in the SUV420H2 purification, is common to HP1γ and HP1β sequences. Moreover, HP1β was identified by Western blotting using specific anti-HP1β antibodies in the TAP-SUV420H2 purified samples (Fig. 1C). Consequently, an interaction between SUV420H2 and HP1β might occur as well. None of the other proteins identified by TAP/MS appeared to be specific to the SUV420H2 purification when compared to our dataset. Thus, we conclude that SUV420H2 mainly interacts with the HP1 isotypes in vivo; HP1α, HP1γ and HP1β as well.

**Figure 2:**
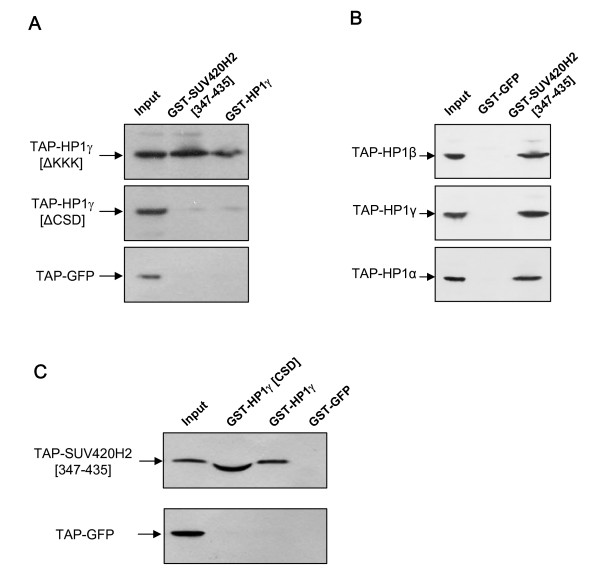
**Identification of SUV420H2 and HP1 interacting domains by in vitro GST-pull down**. (**A**) The heterochromatic targeting module of SUV420H2 interacts with HP1 proteins. GST-tagged truncation SUV420H2 [347–435], as well as control GST-GFP fusion were bound to glutathione-Sepharose and incubated with nuclear extracts from HEK293 cells expressing TAP-tagged HP1α, HP1β, or HP1γ as indicated. After extensive washings, proteins were separated on SDS-PAGE and Western blot probed to reveal TAP-tagged proteins. (**B**) The chromoshadow domain of HP1γ is required for its interaction with SUV420H2. GST-SUV420H2 [347–435], as well as control GST-HP1γ fusions were bound to glutathione-Sepharose and incubated with nuclear extracts from HEK293 cells expressing TAP-HP1γ truncations or TAP-GFP control. HP1γ [ΔKKK] harbours a deletion from lysine 105 to lysine 107 within the hinge region, whereas HP1γ [ΔCSD] contains the 114 N-terminus amino-acids of HP1γ but not the chromoshadow domain. Loss of the chromoshadow domain abolishes the interaction with the heterochromatic targeting module of SUV420H2 and HP1γ oligomerization. (**C**) The chromoshadow domain (CSD) of HP1γ interacts with the SUV420H2 [347–435] region. GST-HP1γ [CSD] consisting of the HP1γ chromoshadow domain (AA 115–183), as well as control GST-HP1γ and GST-GFP fusions were bound to glutathione-Sepharose and incubated with in vitro translated TAP-SUV420H2 [347–435] fusion protein or TAP-GFP control. Bound TAP-tagged proteins are revealed by Western blotting using the peroxidase-anti-peroxidase (PAP) antibody (Sigma) which recognizes the protein A moiety of the TAP tag.

Shotta et al [5] mapped a HP1-binding domain of murine Suv420h2 in the AA349–441 protein region referred as the heterochromatic targeting module, once this domain is also responsible for Suv420h2 targeting at heterochromatin. To confirm that such a functional module is also conserved in the human ortholog, the corresponding region (AA347–435) from human SUV420H2 was expressed in E. coli as a recombinant GST fusion protein and bound to glutathione-Sepharose beads. Full-length TAP-tagged HP1α, HP1β and HP1γ were expressed in HEK293 cells and incubated with GST-SUV420H2 [347–435] fragments. After extensive washings, bound proteins were eluted with glutathione, separated on SDS-PAGE and Western blots were probed with peroxydase coupled anti-peroxydase antibody for detection of TAP-tagged HP1 isotypes. Figure 2A shows that the SUV420H2 [347–435] fragment interacts with all three HP1 proteins. In contrast, recombinant GST-GFP fusion protein does not interact with HP1, excluding unspecific binding to the GST-tag. Thus, the heterochromatic targeting module from human SUV420H2, as previously described for its mouse counterpart [5], interacts with all three HP1 isotypes, HP1α, HP1β and HP1γ . Interestingly, this domain of SUV420H2 contains a PYVRV sequence, which is similar to the pentameric sequences found through a phage display analysis with the Drosophila melanogaster HP1 chromoshadow domain [22] and present in a number of proteins interacting to mammalian HP1 chromoshadow domains [23].

To define the HP1 regions involved in the binding to SUV420H2, TAP-tagged mutants of HP1γ were transiently expressed in HEK293 cells. Nuclear protein extracts were incubated with the recombinant GST-SUV420H2 [347–435] heterochromatic targeting module (Fig. 2B). Deletion of HP1γ chromoshadow domain (CSD) abolishes HP1γ dimerization as expected [24-26], but also the interaction with the SUV420H2 [347–435] fragment, whereas deletion of lysine 105 to lysine 107 within the HP1γ hinge region does not affect binding to the SUV420H2 heterochromatic targeting module. Thus, the HP1 CSD is required for SUV420H2-HP1 interaction. In order to test whether the HP1 CSD is sufficient for such an interaction, a GST-CSD fusion protein was expressed in E. coli, bound to glutathione-Sepharose and incubated with in vitro translated TAP-SUV420H2 [347–435] or TAP-GFP proteins. Figure 2C shows that the SUV420H2 [347–435] fragment specifically interacts with GST-CSD, but not GST-GFP. Taken all together, these results indicate that the interaction between SUV420H2 and HP1 proteins is mediated by the binding of the heterochromatic targeting module to the chromoshadow domain of the respective proteins.

### HP1 is a dynamic component of heterochromatin and its dynamics depend on the cell cycle

**Figure 3:**
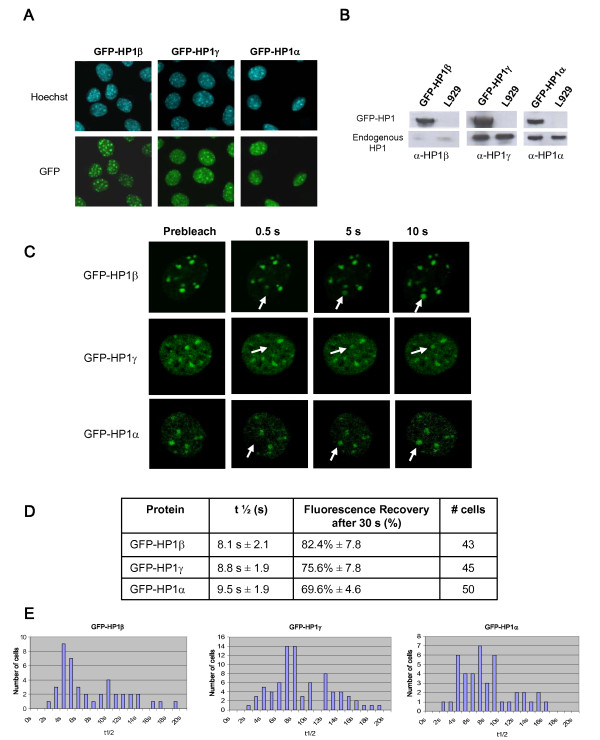
**FRAP analysis of GFP-HP1 shows that HP1 proteins are dynamic components of heterochromatin**. (**A**) Nuclear localization of GFP-tagged proteins. GFP-HP1 expressing L929 cells were fixed with paraformaldehyde and localization of fusion protein was visualized with fluorescence microscopy. Dense foci seen by Hoechst staining represent pericentric heterochromatin regions. (**B**) Detection of GFP-HP1 and endogenous HP1 isoforms in stable cell lines and in L929 cells by Western blotting using specific antibodies for each isoform (α-HP1β, α-HP1γ and α-HP1α). (**C**) Time-lapse series of confocal images of living cells expressing GFP-HP1β, GFP-HP1γ or GFP-HP1α, as indicated. Heterochromatic foci were selected and photobleached. Images were recorded before (Prebleach) and at different time intervals after the bleach. Arrows indicate the photobleached areas. (**B**) Parameters of HP1 protein dynamics from FRAP curves. The half-time of fluorescence recovery (t1/2), the percentage of fluorescence recovery after 30 s, and the number of FRAP curves used are indicated. (**E**) Representation of the number of cells characterized by a given t1/2 value. A single area was photobleached per cell.

To assay for protein dynamics at heterochromatin, fluorescence recovery after photobleaching (FRAP) [27] experiments were performed in live murine L929 cells. These cells were used in our imaging analyses, because mouse fibroblastic cells present a defined distribution of heterochromatic domains revealed by Hoechst staining patterns as bright fluorescent nuclear regions (Fig. 3A).

GFP-HP1 expressing cells were generated using retroviral gene transduction. Fusion proteins of GFP with HP1α, HP1β and HP1γ were shown to localize to these heterochromatin foci (Fig. 3A). The endogenous HP1 counterparts were shown to be localized at the same nuclear domains, as revealed by immunochemistry using specific anti-HP1 antibodies ([28] and data not shown). Expression of GFP-HP1 fusion proteins was analyzed by Western blotting using specific antibodies for each HP1 isotype (Fig. 3B). GFP-HP1 fusion proteins were expressed as full-length proteins at levels significantly higher (GFP-HP1β) or similar (GFP-HP1γ and GFP-HP1α) to those of their endogenous counterparts.

Defined areas of ~1.3 μm of diameter were irreversibly photobleached by five excitation pulses of 336 ms. Fluorescence recovery in the same areas was imaged at regular time intervals (Fig. 3C and see Methods section). The relative fluorescence intensity values for each experiment were collected and plotted in a FRAP curve after correction for photobleaching. The t1/2 value of fluorescence recovery, which is a measure for the speed of replacement of molecules in the bleached area by molecules from the environment was determined, as well as the intensity of fluorescence recovery after 30 s, which is a measure of the mobile fraction of molecules (Fig. 3D and 3E). As previously reported by others in other cell types [14-16,29], Figure 3C–D shows that HP1 proteins are highly dynamic components of heterochromatin in L929 cells. The high mobility of ectopically expressed GFP-HP1 proteins might not be a consequence of protein overexpression since similar observations have been obtained in different experimental settings [14-16,29]. For instances, Schmiedeberg et al [16] determined similar t1/2 values from Hep-2 cells expressing GFP-HP1 proteins at levels lower than those of their endogenous counterparts. It indicates that HP1 dynamic parameters do not depend on the GFP-HP1 expression levels. However, we observed that for each of the three GFP-HP1 isotypes, t1/2 values from one photobleached cell to another show a significantly high variability (Fig. 3E).

**Figure 4:**
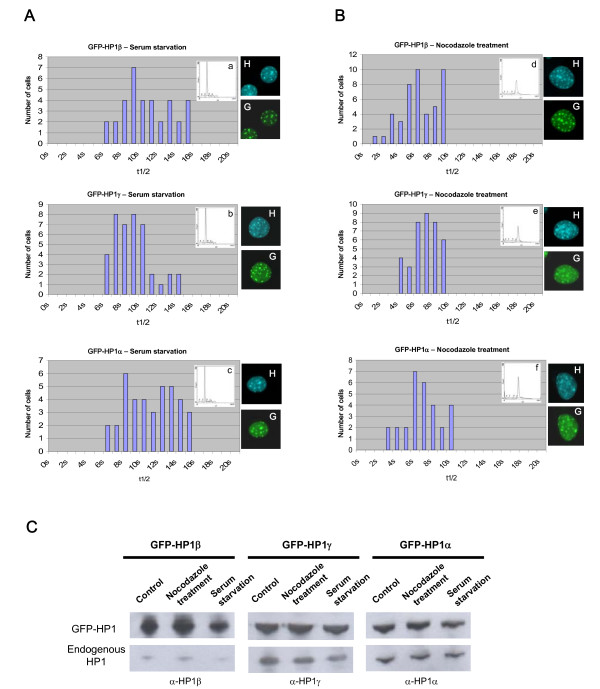
**Dynamics of HP1 varies during cell cycle**. Distribution of t1/2 values deduced from FRAP experiments for GFP-HP1β (upper panels), GFP-HP1γ (middle panels), and GFP-HP1α (lower panels) after serum starvation (**A**) or nocodazole treatment (**B**). The bars indicate the number of cells presenting a given t1/2 value. The effect of the treatments on cell synchronization is measured by FACS analysis (a-f). Nuclear localization of GFP-tagged HP1 proteins (G) and heterochromatin foci revealed by Hoechst staining (H) are shown. (**C**) Detection of GFP-HP1 and endogenous HP1 isoforms by Western blotting using specific antibodies for each isoform (α-HP1β, α-HP1γ and α-HP1α) in absence of treatment (Control), in presence of Nocodazole (50 ng/ml) 24 hours or after 72 hours serum starvation.

In order to test whether this large distribution of t1/2 values could reflect different states of the cells, we performed FRAP experiments after cell synchronization (Fig. 4). GFP-HP1 expressing cells were blocked in G0 by serum starvation for 72 hours, or in G2/M transition using a nocodazole treatment for 24 hours [30]. Cell synchronization was verified by fluorescent-activated cell sorter (FACS) analysis in presence of propidium iodide [31], and FRAP performed. Figure 4A–B shows that, in all cases, cell synchronization reduces the variability of the t1/2 values. After serum deprivation, the t1/2 was ~11.3 s, ~9.4 s, and 11.2 s for HP1α, HP1β and HP1γ, respectively, but only ~7.4 s, ~6.8 s, and ~7.1 s after the nocodazole treatment. Both serum starvation and nocodazole treatment did not affect GFP-HP1 protein localization (Fig. 4A–B) or protein expression (Fig. 4C). This suggests that HP1 proteins at heterochromatin have faster dynamics in G2 rather than in G0 phase of the cell cycle.

**Figure 5:**
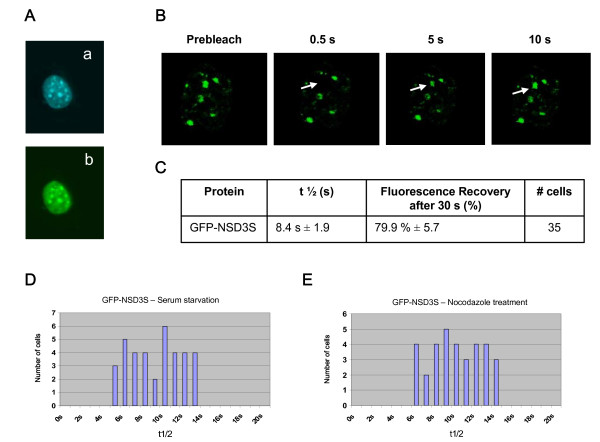
**FRAP analysis of GFP-NSD3S shows that NSD3S is a dynamic component of heterochromatin**. (**A**) Cellular localization of NSD3S. GFP-NSD3S expressing L929 cells were fixed with paraformaldehyde and localization of fusion protein was visualized with fluorescence microscopy. Dense foci seen by Hoechst staining represent pericentric heterochromatin regions (a). GFP-NSD3S labelling co-localizes at these sites (b). (**B**) Time-lapse serie of confocal images of living cells expressing GFP-NSD3S during a FRAP experiment. Images were recorded before (Prebleach) and at different time intervals after the bleach. Arrows indicate the photobleached areas. (**C**) Parameters of NSD3S protein dynamics derived from FRAP curves. The half-time of fluorescence recovery (t1/2), the percentage of fluorescence recovery after 30 s, and the number of FRAP curves used are indicated. (**D**) Representation of the number of cells characterized by a given t1/2 value after cell synchronization by serum starvation. (**E**) Representation of the number of cells characterized by a given t1/2 value after cell synchronization by nocodazole treatment.

In order to demonstrate that these differences in HP1 dynamics were due to intrinsic properties of HP1 but not a consequence of the treatments, similar experiments were performed with NSD3S, another protein associated to heterochromatin. Based on alternative splicing events, the NSD3 gene encodes two different proteins [32]: a long isoform (NSD3L) related to the histone methyltransferase Nsd1, and a short isoform (NSD3S) which does not contain the SET domain and the PHD fingers, but still harbouring a PWWP motif. NSD3S was stably expressed as a GFP fusion protein in L929 cells using retroviral transduction. Figure 5A shows that NSD3S is targeted at pericentric heterochromatin. FRAP experiments revealed that NSD3S is as dynamic as HP1 at heterochromatin (Fig. 5B–C). GFP-NSD3S fluorescence recovery reached ~80% after 30 s with a t1/2 value ~8.4 s. These dynamic parameters are similar to those of HP1 proteins (Fig. 3D), indicating that HP1 proteins are not the only mobile components associated to heterochromatin with such a highly dynamic exchange rate. Next, FRAP experiments after cell synchronization were performed on GFP-NSD3S expressing L929 cells. Figure 5D–E shows that t1/2 values are relatively similar after serum starvation and nocodazole treatment. This result indicates that the treatments used to induce cell synchronization do not modify significantly NSD3S dynamics at heterochromatin. Consequently, the differences of mobility in HP1 protein dynamics during the cell cycle are indeed due to their intrinsic properties, but not to culture conditions.

### SUV420H2 is a stable component of heterochromatin

**Figure 6:**
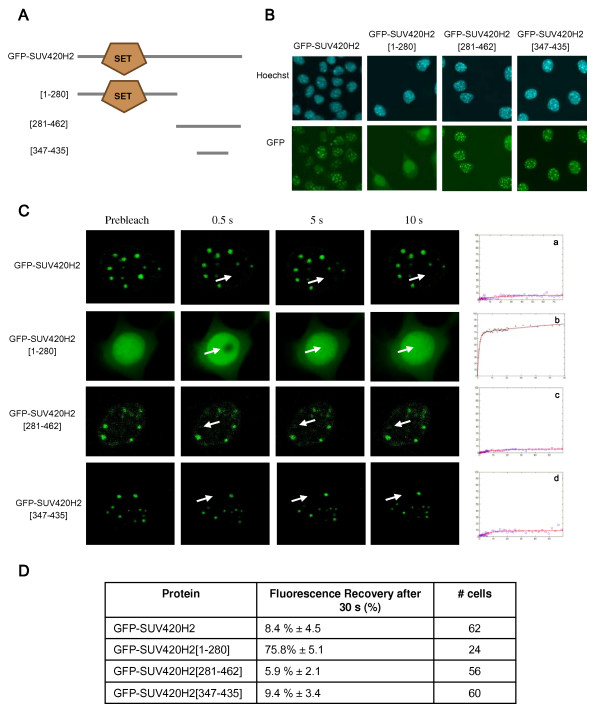
**FRAP analysis of GFP-SUV420H2 shows that SUV420H2 is strongly associated to heterochromatin**. (**A**) Schematic representation of various SUV420H2 truncations used. The catalytically active SET domain is indicated. (**B**) Cellular localization of SUV420H2 and the truncations expressed as GFP fusion proteins in L929 cells. Cells were fixed with paraformaldehyde and localization of fusion proteins was visualized with fluorescence microscopy. Dense foci seen by Hoechst staining represent pericentric heterochromatin regions (upper panels). GFP labelling is shown (lower panels). (**C**) Time-lapse series of confocal images of living cells expressing GFP-SUV420H2, GFP-SUV420H2 [1–280], GFP-SUV420H2 [281–462], and GFP-SUV420H2 [347–435] as indicated, during FRAP experiments. Images were recorded before (Prebleach) and at different time intervals after the bleach. Arrows indicate the photobleached areas. Relative fluorescence intensities are displayed in recovery curves (a-d). (**D**) Protein and truncations dynamics derived from FRAP curves. The percentage of fluorescence recovery after 30 s, and the number of FRAP curves used are indicated.

**Figure 7:**
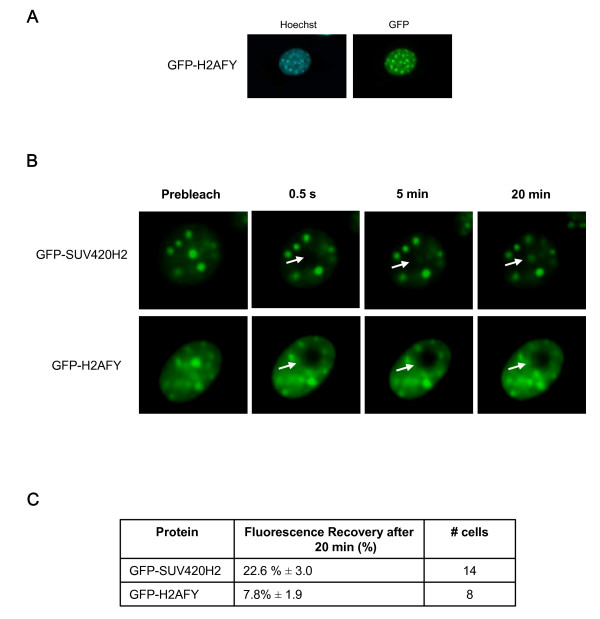
**Comparison of SUV420H2 and H2AFY dynamics by FRAP**. (**A**) Cellular localization of GFP-H2AFY fusion proteins stably expressed in L929 cells. Cells were fixed with paraformaldehyde and localization of fusion proteins was visualized with fluorescence microscopy. GFP-H2AFY fusion proteins colocalize with dense foci identified by Hoechst staining as pericentric heterochromatin regions. (**B**) Time-lapse series of confocal images of living cells expressing GFP-SUV420H2 and GFP-H2AFY as indicated, during FRAP experiments. Images were recorded before (Prebleach) and at different time intervals after the bleach. Arrows indicate the photobleached areas. (**C**) Protein dynamics derived from FRAP curves. The percentage of fluorescence recovery after 20 min, and the number of FRAP curves used are indicated.

To gain insights into the dynamic properties of SUV420H2 and its association to heterochromatin, FRAP experiments were performed on L929 stably expressing a GFP-SUV420H2 fusion protein (Fig. 6C–D). In contrast to HP1 proteins, GFP-SUV420H2 fluorescence recovery reached only 10% as a maximum after 60 s, suggesting that ~90% of the SUV420H2 fraction is stably bound within heterochromatic domains. Even after several minutes, the intensity of fluorescence recovery remains weak (see below, Figure 7). Together, these results indicate that SUV420H2 is more stably associated to heterochromatin than HP1 proteins. This suggests that in addition to its function as a histone methyltransferase, SUV420H2 might have a structural role in chromatin organization.

To identify the protein domains involved in stable association of SUV420H2 at heterochromatin, we stably expressed various protein deletions as GFP fusions in L929 cells. Figure 6A shows a schematic representation of the protein truncations used. Consistent with previous data [5], the N-terminus of SUV420H2 comprising the catalytically active SET domain is dispersed within the nucleus, whereas the C-terminal part of the protein is associated with heterochromatin that corresponds to Hoechst dense staining (Fig. 6B). Moreover, the 88 amino-acids region comprising the heterochromatic targeting module of SUV420H2 is sufficient to recapitulate full length protein localization. Next, FRAP studies were performed in L929 cells expressing SUV420H2 C-termini-GFP fusion proteins still located at heterochromatin foci. The C-terminal truncation as well as the heterochromatic targeting module alone, show kinetic properties similar to those of SUV420H2 full length (Fig. 6C–D). At the opposite, the SUV420H2 [1–280] protein region comprising the SET domain, is highly mobile. Thus, in contrast to SUV39H1 for which the SET domain has been shown to contribute to heterochromatin binding [17], all the dynamic characteristics of SUV420H2 are restricted to the 88 amino-acids heterochromatic targeting module.

So far, the mechanism by which the heterochromatic targeting module is tightly bound to chromatin remains elusive. Using a GST-pull down-mass spectrometry strategy, the only proteins found to interact in vitro with this domain were the HP1 proteins (data not shown), but other approaches are ongoing to unravel the molecular mechanisms involved in the linkage of SUV420H2 to heterochromatin.

Next, we compared the SUV420H2 kinetics with the one of H2AFY. H2AFY (macroH2A1) is a variant of histone H2A, enriched at the inactive X chromosome in female mammals and found at other chromosomal locations [33]. Since H2AFY is more resistant to salt extraction than canonical H2A [34], it is expected that H2AFY might be in more stable association with H3-H4 and/or DNA than H2A. H2AFY was stably expressed as a GFP fusion protein in L929 cells using retroviral transduction. Figure 7A shows that H2AFY is targeted at foci identified by Hoechst staining as pericentric heterochromatin regions, like SUV420H2. FRAP experiments performed over more than 30 min showed that fluorescence recovery after 20 min is ~22% and ~7% for GFP-SUV420H2 and GFP-H2AFY, respectively (Fig. 7B–C). Thus, SUV420H2 is stably bound to heterochromatin, but not as strongly as the nucleosome component, H2AFY.

## Conclusion

The SUV420H family of histone methyltransferases catalyzes trimethylation of histone H4K20 which is characteristic to pericentric heterochromatin. So far, little is known about the mechanisms by which this protein class is recruited to and interacts with chromatin.

Here, we applied the TAP/MS approach in order to identify proteins associated to the histone methyltransfase SUV420H2. In our purification, a relatively weak amount of SUV420H2 was recovered, presumably because the protein is tightly bound to chromatin. We assume that the SUV420H2 captured by TAP mainly correspond to the nucleoplasm soluble fraction. Most of the proteins identified by TAP/MS appeared to be nonspecific and recovered in a number of unrelated purifications (data not shown). However, members of the HP1 family were found as specific SUV420H2-binding partners. Since only part of the SUV420H2 population was purified, we cannot exclude that a number of SUV420H2 interacting partners, and in particular the proteins associated to the SUV420H2 fraction the most tightly bound to chromatin, were not identified.

We used in vitro GST-pull down assays, to map the domains of interaction between SUV420H2 and HP1 proteins. As previously described for the mouse Suv420h2 ortholog [5], an 88 amino-acids region defined as the heterochromatic targeting module of SUV420H2 interacts with HP1α, HP1β and HP1γ proteins. Using deletion mutants, the HP1 chromoshadow domain was shown to be sufficient for binding to the SUV420H2 heterochromatic targeting module.

To investigate the mechanisms by which SUV420H2 could be recruited at heterochromatin and the possible role of HP1 proteins in this recruitment, protein dynamics were studied using fluorescence recovery after photobleaching (FRAP) in living cells [27]. For a long time, heterochromatin was considered as a stable structure maintained in an inaccessible conformation that would exclude transcriptional activation. This concept has changed when HP1 was shown by FRAP analysis to be highly mobile [14,15]. In our study, we show that NSD3S is another protein transiently bound to pericentric heterochromatin, reinforcing the idea that the dynamic nature of heterochromatin is essential to its function. Along our study, we also showed that HP1 exchange kinetics is modified during the cell cycle, indicating that heterochromatin organization is modulated during the cell cycle progression. However, not all the proteins bound to pericentric heterochromatin are in continuous flux with the nucleoplasm since Krouels et al. showed that a substantial fraction of the histone methyltransferase SUV39H1 is stably associated with chromatin [17]. In this study, we demonstrated that the major part of SUV420H2 is also tightly bound at pericentric heterochromatin, indicating that in addition to its histone methyltransferase activity, SUV420H2 might have a structural role in chromatin organization. We showed that the heterochromatic targeting module is sufficient to fully recapitulate SUV420H2 dynamic properties. Then, in contrast to SUV39H1 [17], the SET domain of SUV420H2 is not required for stable binding of the protein to pericentric heterochromatin.

Genetic studies indicated that H3K9 trimethylation would precede H4K20 trimethylation, and that HP1 which recognizes H3K9 trimethylation could target SUV420H class of histone methyltransferases to heterochromatin [5]. Our data confirm such an interaction in vitro and in vivo. However, FRAP experiments reveal that in contrast to HP1, SUV420H2 is strongly associated to pericentric heterochromatin. Thus, the fraction of SUV420H2 captured and characterized by TAP/MS is the soluble fraction which may be in a stable association with HP1. Consequently, we speculate that SUV420H2 is recruited to heterochromatin in association with HP1, but stably maintained at its sites in an HP1-independent fashion.

## Methods

### Constructs and vectors engineering

Full-length open reading frames of all cDNAs, as well as truncated and mutant cDNAs were PCR amplified from IMAGE cDNA clones purchased from Deutsches Ressourcenzentrum für Genomforschung GmbH (RZPD) and cloned into the GATEWAY entry vector pDONR201 (Invitrogen).

Moloney murine leukemia virus-based vectors were generated from pLNCX2 (Clontech) in which an IRES-pac cassette from pIRESpuro (Clontech) and conferring resistance to puromycin was introduced. Then, GATEWAY compatible target vectors for expression of TAP-tagged (pRP-NTAP-GW) or GFP-tagged (pRP-NGFP-GW) proteins were engineered by inserting GATEWAY and TAP (from pZome-1-N, Cellzome) or GFP (from pEGFP-N1, Clontech) cassettes, respectively. For bacterial expression of the GST-tagged proteins, a GATEWAY cassette was introduced into pGEX-4T1 (Amersham). Entry clones were recombined into suitable target vectors by GATEWAY LR reactions. A list of clones used, primer and vector sequences is available on request.

### Cell culture and stable cell lines

HeLa (Human cervix epitheloid carcinoma – ECACC), L929 (Mouse cell line C3H/An connective tissue – ECACC) and HEK293 (ECACC) cell lines were maintained in Dulbecco's modified Eagle's medium (DMEM) supplemented with 10% fetal bovine serum (Invitrogen), 100 U/ml penicillin (Invitrogen), and 100 μg/ml streptomycin (Invitrogen) at 37°C in humidity-saturated 5% CO_2 _atmosphere.

Retroviral stable cell lines were generated according to the following procedure. Phoenix amphotropic packaging cells (3 × 10^5 ^cells/well) were seeded in a 6-well plate and transfected 24 hours later with 0.8 μg of retroviral plasmid using Exgen 500 (Euromedex) following the instructions of the manufacturer. After 48 hours virus-containing supernatant was filtered through a 0.45-μm-pore-size filter. HeLa or L929 cells (10^5 ^each) were seeded in a six-well plate and transduced with 3 ml filtered virus supernatant in the presence of 8 μg/ml of Polybrene for an infectious round of 24 hours. Cells were then incubated for 24 hours in normal medium. The polyclonal population of cells was then selected with 1 μg/ml of puromycin. Growing cells were then tested for recombinant protein expression using immunocytochemistry for TAP-tagged protein expression or immunofluorescence for GFP-tagged protein expression.

For cell cycle synchronization, cells were seeded at a concentration of 2 × 10^4 ^cells/cm^2 ^either in 6-well plates or in 10-cm dishes and grown to 50–70% confluence to obtain cultures in the logarithmic growth phase. Synchronization in G0 was achieved by serum deprivation; cultures were washed 3 times with PBS, once with DMEM and then cultured for 72 hours in DMEM without serum, whereas synchronization at G2/M transition was achieved using a nocodazole treatment [30]; cultures were grown in DMEM with 10% fetal bovine serum and 50 ng/ml nocodazole for 24 hours. All synchronization experiments based on serum deprivation or nocodazole treatment were performed in parallel to ensure accurate comparisons.

### Antibodies and Western blotting

The following primary antibodies were used for Western blotting: mouse anti-HP1α antibody (2HP-2G9, Euromedex; used at a dilution of 1:2 000), mouse anti-HP1β antibody (1MOD-1A9, Euromedex; used at a dilution of 1:2 000), mouse anti-HP1γ antibody (2MOD-1G6, Euromedex; used at a dilution of 1:2 000), goat anti-CBP antibody (sc-9456, Santa Cruz Biotechnology; used at a dilution of 1:300). Secondary antibodies were: horseradish peroxidase-congugated goat anti-mouse antibody (115-035-003, Jackson ImmunoResearch; used at a dilution of 1:10 000), horseradish peroxidase-conjugated donkey anti-goat antibody (705-035-003, Jackson ImmunoResearch; used at a dilution of 1:5 000). The protein A moiety of the TAP tag was revealed with rabbit peroxidase anti-peroxidase antibody (P1291, Sigma; used at a dilution of 1:10 000).

For Western blotting, protein samples (50 μg) in SDS loading buffer were electrophoresed on 4–12% Bis-Tris gels (Invitrogen) and transferred to nitrocellulose membranes (Schleicher & Schuell). The membranes were blocked in 10% milk powder in PBS-T (1× PBS with 0.1% Tween20) for 1 hour at room temperature, incubated for same time with the primary antibody in PBS-T, and washed three times 10 min in PBS-T. The membranes were then incubated with the peroxidase-conjugated secondary antibody in PBS-T for 1 hour and afterward washed three times 10 min in PBS-T. Signal was detected using chemiluminescence reagent (ECL, Amersham) on imaging film (GE Healthcare).

### Flow cytometry

Cell cycle analysis was performed by flow cytometry as described by Evans et al [31]. Single-cell suspensions were obtained by trypsinization, washed twice with PBS and incubated in 80% ethanol at room temperature for 16–24 hours. Cells were rinsed with PBS, resuspended in 200 μg/ml Propidium Iodide (PI), incubated with 100 μg/ml RNase at 37°C for 30 min, and stored at least one hour at 4°C. Stained cells were analyzed with a Coulter Epics XL flow cytometer equipped with a 488-nm argon laser (Beckman Coulter, Fullerton, CA) and a 530 nm band-pass filter allowing detection of PI fluorescence. A minimum of 10,000 events were collected. GFP or PI fluorescence was expressed as a ratio of the mean channel value of the GFP or PI histogram to the mean channel value of the isotype control histogram.

### Tandem affinity purification

For Tandem affinity purification (TAP), cells expressing a TAP-tagged protein were expanded into forty 15-cm dishes. At confluence (about 100 mg total protein), cells were harvested, washed with PBS, resuspended in cell lysis buffer (10 mM Tris-HCl pH 7.4, 1.5 mM MgCl_2_, 10 mM KCl, 25 mM NaF, 1 mM Na_3_VO_4_, 1 mM dithiothreitol [DTT] and complete protease inhibitors [Roche]) and homogenized by 20 strokes with a type B pestle. Nuclei were recovered by centrifugation 10 min at 2000 g, resupended in nuclear lysis buffer (50 mM Tris-HCl pH 7.4, 1.5 mM MgCl_2_, 420 mM NaCl, 20% glycerol, 25 mM NaF, 1 mM Na_3_VO_4_, 1 mM dithiothreitol [DTT] and complete protease inhibitors [Roche]), and then incubated for 1 hour in a rotation wheel at 4°C to extract nuclear proteins. Lysates were subsequently clarified by ultracentrifugation at 100,000 g, 1 hour.

Nuclear lysate was incubated with IgG agarose beads (Sigma) for 2 hours at 4°C in a rotation wheel. Bound proteins were washed with an excess of lysis buffer and then with a TEV-protease cleavage buffer (10 mM Tris-HCl pH 7.5, 100 mM NaCl and 0.2% NP-40) and eluted by addition of 30 mg TEV protease (Invitrogen) for 2 hours at 16°C. The TEV-protease cleavage product was incubated with calmodulin sepharose (Amersham) in the presence of 2 mM CaCl_2 _for 30 min at 4°C in a rotation wheel. After extensive washes in 100 mM Trs-HCl pH8, 100 mM NaCl, 0.5 mM EDTA, 2 mM CaCl_2_, calmodulin-bound proteins were eluted by boiling in SDS loading buffer.

### Mass spectrometric analysis

Protein eluate was separated on a 4–12% NuPAGE Novex gel (Invitrogen) and stained with Imperial Protein Stain (Pierce). Gel was sliced into 37 bands across the entire separation range of the lane. Cut bands were reduced, alkylated with iodoacetamide, and in-gel digested with trypsin (Promega) as described previously [35]. In brief, gel bands were destained overnight at 4°C in a solution containing 50 mM NH_4_HCO_3 _and 50% acetonitrile, dehydrated in acetonitrile, and dried in a vacuum centrifuge. Gel pieces were then rehydrated at 4°C for 45 min in a digestion buffer (25 mM NH_4_HCO_3 _and 12.5 ng/μl trypsin). The supernatant was replaced by 50 μl of 25 mM NH_4_HCO_3_, and the samples were incubated overnight at 37°C. The tryptic peptides were recovered by 10-min incubations, twice in 45% acetonitrile, 10% HCOOH and once in 95% acetonitrile, 5% HCOOH. All supernatants were pooled and dried in a vacuum centrifuge.

Each tryptic digest sample was subjected to nano-LC-nano-ESI-MS/MS analysis on an ion trap mass spectrometer (LCQ Deca XP^+^, Thermo Electron Corp.), equipped with a nanoelectrospray ion source, coupled with a nano-high pressure liquid chromatography system (LC Packings Dionex). Samples were resuspended in 3 μl of 0.1% HCOOH, and 1.4 μl were injected into the mass spectrometer using a Famos autosampler (LC Packings Dionex). The samples were first desalted and then concentrated on a reverse phase precolumn of 5 mm × 0.3 mm inner diameter (Dionex) by solvent A (95% H_2_O, 5% acetonitrile, 0.1% HCOOH) delivered by the Switchos pumping device (LC Packings Dionex) at a flow rate of 10 μl/min for 3 min. Peptides were separated on a 15 cm × 75 μm-inner diameter C18 PepMap column (Dionex). The flow rate was set at 200 nl/min. Peptides were eluted using a 5–70% linear gradient of solvent B (20% H_2_O, 80% acetonitrile, 0.08% HCOOH) in 45 min. Coated nanoelectrospray needles (360 μm outer diameter, 20 μm inner diameter, 10 μm tip inner diameter, standard coating) were obtained from New Objective (Woburn, MA). Spray voltage was set at 1.5 kV, and capillary temperature was set at 170°C. The mass spectrometer was operated in positive ionization mode.

Data acquisition was performed in a data-dependent mode consisting of, alternatively in a single run, a full-scan MS over the range m/z 500–2,000 and a full MS/MS of the ion selected in an exclusion dynamic mode (the most intense ion is selected and excluded for further selection for a duration of 3 min). MS/MS data were acquired using a 2 m/z unit ion isolation window and 35% relative collision energy. MS/MS .raw data files were transformed to .dta files with the Bioworks 3.1 software (Thermo Electron Corp.). The .dta files generated were next concatenated with merge.bat (a DOS batch file for Windows) to be uploaded in Mascot public interface version 2.2.03  for database searches in Swiss-Prot 55.1 (359,942 sequences; 129,199,355 residues).

Search parameters in human sequences were: three allowed missed cleavages, methionine oxidation and cysteine carbamidomethylation as variable modifications, 2 Da for peptide tolerance, and 0.8 Da for MS/MS tolerance. Results were scored using the probability-based Mowse score [the protein score is -10 × log(p), where p is the probability that the observed match is a random event]. Most proteins were unambiguously identified by the sequencing of several independent peptides. Identifications with Mascot individual ion score < 38 or with the significance threshold p > 5% (indicate identity or extensive homology) were categorically rejected. In addition, because a shared sequence may represent a problem, for single peptide identifications, all sequences obtained by MS/MS analysis were checked using the Basic Local Alignment Search Tool (BLAST) public interface (version 2.2.18) to exclude that sequence sharing with other proteins could interfere with the reliability of the identification.

### In vitro GST protein binding assays

GST fusion proteins, were expressed in E. coli BL21 (DE3) and purified on glutathione-Sepharose 4B (GE Healthcare) according to the manufacturer's instructions. GST-proteins were then fixed on glutathione-Sepharose 4B and stored in STE buffer (10 mM Tris-HCl pH8, 150 mM NaCl, 1 mM EDTA and complete protease inhibitors [Roche]). After preclearing with empty beads, nuclear extracts (~500 μg proteins) were incubated with immobilized GST-fusion proteins overnight at 4°C. Beads were washed four times with E1A Buffer (50 mM Hepes, pH 7.9, 1 mM EGTA, 250 mM NaCl, 1 mM DTT, 1 mM EDTA and complete protease inhibitors [Roche]) and bound proteins were recovered with the Elution Buffer (10 mM glutathione in 50 mM Tris-HCl pH 8.0), resolved by 4–12% gradient SDS-PAGE (Invitrogen) and visualized by Western blotting using the peroxidase-anti-peroxidase (PAP) antibody (Sigma) which recognizes the protein A moiety of the TAP tag. Input material corresponds to 2% of the material used in the binding assays.

Nuclear extracts used for GST-pull downs were prepared from HEK293 cells transiently expressing TAP-tagged proteins. Forty eight hours after transfection, cells were lysed in low salt buffer (10 mM Tris-HCl pH 7.4, 25 mM NaCl, 2 mM MgOAc, 1 mM DTT, 1 mM EDTA, 0.05% NP-40 and complete protease inhibitors [Roche]) for 15 min at 4°C. Nuclei were pelleted by centrifugation at 4°C for 10 min at 800 g, and lysed in E1A buffer during 2 hours by gently shaking at 4°C. Nuclear proteins were recovered by centrifugation at 2500 g, 10 min at 4°C.

In vitro translated proteins used for GST-pull downs were produced with the TnT7 Quick Coupled Transcription/Translation System (Promega). Immobilized GST-fusion proteins were incubated with in vitro translated proteins overnight at 4°C in IP buffer (50 mM Tris-HCl pH7.5, 500 mM NaCl, 1 mM EDTA, 0.5% NP-40, 10% glycerol and complete protease inhibitors [Roche]). The beads were washed four times with IP buffer and resuspended in loading buffer. Bound proteins were resolved by SDS-PAGE and visualized by Western blotting.

### Fluorescence recovery after photobleaching

FRAP experiments were performed in living cells and dynamic parameters determined according to McNally [36]. Briefly, GFP-expressing L929 cells were plated on coverslip surfaces, grown in L15 medium (Invitrogen), and maintained at 37°C in a Leica live-cell chamber. A Leica SP2 confocal microscope equipped with an oil 100× NA 1.4 plan Apo lens objective, a 488 nm mono-ray laser line, and a linear regime detector in intensity measurements were used for FRAP experiments [37]. Five pre-bleach acquisitions were performed to measure background, fluorescence fading, and pre-bleach fluorescence intensity. The laser power was calibrated using an acousto-optical tunable filter (AOTF). Pre-bleach and post-bleach imaging were performed with an AOTF setting at 5% (5 mW), whereas photobleaching was at 100% AOTF (100 mW). The bleached area was chosen circular for simplicity of the equations available to analyze fluorescence recovery for such a shape [38]. The bleached regions had a size of 1 to 1.3 μm of diameter, and were subjected to five excitation pulses of 336 ms each at high laser power. Post-bleach images were collected at 20 time intervals of 336 ms, 15 time intervals of 1,336 s and 15 time intervals of 2,336 s.

Relative fluorescence intensities within the bleached area were plotted as a function of time, yielding the RAW FRAP curve. The recovery curves were corrected for background, fluorescence fading, and decrease in fluorescence during photobleaching. The t1/2 value was defined as the time required for reaching half-maximum recovery and was calculated from the corrected recovery curves obtained using an in-house modified MATLAB fitting tool (Mathworks) using fit curves where the intensity recovered in time follows the exponential law,



according to McNally and colleagues [36,39].

## Abbreviations

TAP: Tandem Affinity Purification; MS: Mass Spectrometry; FRAP: Fluorescence Recovery After Photobleaching; AA: amino-acid; CSD: Chromoshadow domain; TEV protease: Tobacco etch virus protease

## Authors' contributions

PPS performed FRAP experiments and analyses. PV carried out molecular clonings, stable cell line generation, and participated to TAP experiments. DT participated to FRAP curve analyses. JV participated to TAP and performed MS analyses. CR carried out GST-pull down and FRAP experiments. LH participated in the coordination of the FRAP experiments. POA conceived, designed and coordinated the study, and wrote the manuscript. All authors read and approved the final manuscript.

## Supplementary Material

Additional file 1 **List of proteins identified with SUV420H2**. Proteins identified by LC-MS/MS in the SUV420H2 tandem affinity purification are listed. Click here for file

Additional file 2 **List of peptides identified by TAP with SUV420H2**. For each protein, the sequences of the peptides identified by LC-MS/MS in the SUV420H2 tandem affinity purification are listed. Click here for file
