# Supplementary material for: The histone methyltransferase SUV420H2 and Heterochromatin Proteins HP1 interact but show different dynamic behaviours
Source: BMC Cell Biol. 2009 Jun 1;10:41. doi: 10.1186/1471-2121-10-41 (PMC2701926; doi:10.1186/1471-2121-10-41)
Supplement: Additional file 1 — List of proteins identified with SUV420H2. Proteins identified by LC-MS/MS in the SUV420H2 tandem affinity purification are listed. [file 1471-2121-10-41-S1.pdf]

## List of proteins identified with SUV420H2

TAP bait is indicated in bold

| Accession | Description | Protein Score | Number of Peptides |
|---|---|---|---|
| 5HT7R_HUMAN | 5-hydroxytryptamine receptor 7 | 39 | 3 |
| A26CA_HUMAN | ANKRD26-like family C member 1A - Homo sapiens (Human) | 155 | 4 |
| ACTB_HUMAN | Actin, cytoplasmic 1 - Homo sapiens (Human) | 534 | 17 |
| ACTBL_HUMAN | Protein beta-actin-like - Homo sapiens (Human) | 136 | 7 |
| ACTC_HUMAN | Actin, alpha cardiac muscle 1 - Homo sapiens (Human) | 326 | 16 |
| ADT2_HUMAN | ADP/ATP translocase 2 - Homo sapiens (Human) | 45 | 1 |
| CALM_HUMAN | Calmodulin - Homo sapiens (Human) | 101 | 3 |
| CBX3_HUMAN | Chromobox protein homolog 3 - Homo sapiens (Human) | 422 | 20 |
| CBX5_HUMAN | Chromobox protein homolog 5 - Homo sapiens (Human) | 46 | 3 |
| CH60_HUMAN | 60 kDa heat shock protein, mitochondrial precursor - Homo sapiens (Human) | 132 | 3 |
| CPNS1_HUMAN | Calpain small subunit 1 - Homo sapiens (Human) | 160 | 8 |
| DHX9_HUMAN | ATP-dependent RNA helicase A - Homo sapiens (Human) | 42 | 1 |
| DNJA1_HUMAN | DnaJ homolog subfamily A member 1 - Homo sapiens (Human) | 132 | 2 |
| DYH12_HUMAN | Dynein heavy chain 12, axonemal | 38 | 2 |
| EF1A1_HUMAN | Elongation factor 1-alpha 1 - Homo sapiens (Human) | 160 | 4 |
| EFTU_HUMAN | Elongation factor Tu, mitochondrial precursor - Homo sapiens (Human) | 276 | 4 |
| GRP78_HUMAN | 78 kDa glucose-regulated protein precursor - Homo sapiens (Human) | 398 | 6 |
| GTF2I_HUMAN | General transcription factor II-I - Homo sapiens (Human) | 85 | 1 |
| H2A1D_HUMAN | Histone H2A type 1-D - Homo sapiens (Human) | 126 | 4 |
| H2B1C_HUMAN | Histone H2B type 1-C/E/F/G/I - Homo sapiens (Human) | 123 | 3 |
| H31T_HUMAN | Histone H3.1t - Homo sapiens (Human) | 134 | 8 |
| H4_HUMAN | Histone H4 - Homo sapiens (Human) | 152 | 7 |
| HNRL2_HUMAN | Heterogeneous nuclear ribonucleoprotein U-like protein 2 - Homo sapiens (Human) | 67 | 1 |
| HNRPU_HUMAN | Heterogeneous nuclear ribonucleoprotein U - Homo sapiens (Human) | 136 | 2 |
| HS90A_HUMAN | Heat shock protein HSP 90-alpha - Homo sapiens (Human) | 55 | 1 |
| HS90B_HUMAN | Heat shock protein HSP 90-beta - Homo sapiens (Human) | 69 | 1 |
| HSP7C_HUMAN | Heat shock cognate 71 kDa protein - Homo sapiens (Human) | 408 | 6 |
| IQGA1_HUMAN | Ras GTPase-activating-like protein IQGAP1 - Homo sapiens (Human) | 70 | 1 |
| K1967_HUMAN | Protein KIAA1967 - Homo sapiens (Human) | 83 | 2 |
| K6PP_HUMAN | 6-phosphofructokinase type C - Homo sapiens (Human) | 220 | 4 |
| KU70_HUMAN | ATP-dependent DNA helicase 2 subunit 1 - Homo sapiens (Human) | 51 | 1 |
| KU86_HUMAN | ATP-dependent DNA helicase 2 subunit 2 - Homo sapiens (Human) | 71 | 1 |
| LHX1_HUMAN | LIM/homeobox protein Lhx1 - Homo sapiens (Human) | 41 | 2 |
| MATR3_HUMAN | Matrin-3 - Homo sapiens (Human) | 120 | 4 |
| MCM7_HUMAN | DNA replication licensing factor MCM7 - Homo sapiens (Human) | 145 | 3 |
| MFAP4_HUMAN | Microfibril-associated glycoprotein 4 precursor - Homo sapiens (Human) | 75 | 3 |
| MPCP_HUMAN | Phosphate carrier protein, mitochondrial precursor - Homo sapiens (Human) | 44 | 1 |
| MYH14_HUMAN | Myosin-14 | 68 | 1 |
| MYH9_HUMAN | Myosin-9 - Homo sapiens (Human) | 589 | 10 |
| MYLK2_HUMAN | Myosin light chain kinase 2, skeletal/cardiac muscle - Homo sapiens (Human) | 70 | 6 |
| OFD1_HUMAN | Oral-facial-digital syndrome 1 protein | 40 | 2 |
| PP1A_HUMAN | Serine/threonine-protein phosphatase PP1-alpha catalytic subunit - Homo sapiens (Human) | 58 | 1 |
| PYR1_HUMAN | CAD protein [Includes: Glutamine-dependent carbamoyl-phosphate synthase - Homo sapiens (Human) | 202 | 4 |
| RL11_HUMAN | 60S ribosomal protein L11 - Homo sapiens (Human) | 82 | 1 |
| RL12_HUMAN | 60S ribosomal protein L12 - Homo sapiens (Human) | 68 | 1 |
| RL23_HUMAN | 60S ribosomal protein L23 - Homo sapiens (Human) | 84 | 1 |
| RL23A_HUMAN | 60S ribosomal protein L23a - Homo sapiens (Human) | 80 | 1 |
| RL27A_HUMAN | 60S ribosomal protein L27a - Homo sapiens (Human) | 76 | 2 |
| RL30_HUMAN | 60S ribosomal protein L30 - Homo sapiens (Human) | 58 | 1 |
| RLA0_HUMAN | 60S acidic ribosomal protein P0 - Homo sapiens (Human) | 104 | 3 |
| RLA1_HUMAN | 60S acidic ribosomal protein P1 - Homo sapiens (Human) | 58 | 1 |
| RS11_HUMAN | 40S ribosomal protein S11 - Homo sapiens (Human) | 83 | 2 |
| RS13_HUMAN | 40S ribosomal protein S13 - Homo sapiens (Human) | 75 | 2 |
| RS15A_HUMAN | 40S ribosomal protein S15a - Homo sapiens (Human) | 48 | 2 |
| RS17_HUMAN | 40S ribosomal protein S17 - Homo sapiens (Human) | 65 | 1 |
| RS18_HUMAN | 40S ribosomal protein S18 - Homo sapiens (Human) | 78 | 2 |
| RS25_HUMAN | 40S ribosomal protein S25 - Homo sapiens (Human) | 50 | 2 |
| RS3_HUMAN | 40S ribosomal protein S3 - Homo sapiens (Human) | 333 | 7 |
| RS3A_HUMAN | 40S ribosomal protein S3a - Homo sapiens (Human) | 49 | 1 |
| RS4Y1_HUMAN | 40S ribosomal protein S4, Y isoform 1 - Homo sapiens (Human) | 56 | 1 |
| RS5_HUMAN | 40S ribosomal protein S5 - Homo sapiens (Human) | 62 | 1 |
| S10A2_HUMAN | Protein S100-A2 - Homo sapiens (Human) | 86 | 3 |
| SFPQ_HUMAN | Splicing factor, proline- and glutamine-rich - Homo sapiens (Human) | 70 | 1 |
| SP16H_HUMAN | FACT complex subunit SPT16 - Homo sapiens (Human) | 68 | 1 |
| SPG7_HUMAN | Paraplegin - Homo sapiens (Human) | 41 | 2 |
| SPTA2_HUMAN | Spectrin alpha chain, brain - Homo sapiens (Human) | 95 | 2 |
| SSB_HUMAN | Single-stranded DNA-binding protein, mitochondrial precursor - Homo sapiens (Human) | 68 | 1 |
| **SUV42_HUMAN** | **Histone-lysine N-methyltransferase SUV420H2 - Homo sapiens (Human)** | **587** | **20** |
| TBA1A_HUMAN | Tubulin alpha-1A chain - Homo sapiens (Human) | 61 | 2 |
| TBA1B_HUMAN | Tubulin alpha-1B chain - Homo sapiens (Human) | 679 | 16 |
| TBA1C_HUMAN | Tubulin alpha-1C chain - Homo sapiens (Human) | 512 | 23 |
| TBA4A_HUMAN | Tubulin alpha-4A chain - Homo sapiens (Human) | 656 | 16 |
| TBA8_HUMAN | Tubulin alpha-8 chain - Homo sapiens (Human) | 306 | 8 |
| TBB1_HUMAN | Tubulin beta-1 chain - Homo sapiens (Human) | 211 | 11 |
| TBB2A_HUMAN | Tubulin beta-2A chain - Homo sapiens (Human) | 896 | 26 |
| TBB2C_HUMAN | Tubulin beta-2C chain - Homo sapiens (Human) | 1114 | 31 |
| TBB3_HUMAN | Tubulin beta-3 chain - Homo sapiens (Human) | 650 | 23 |
| TBB4_HUMAN | Tubulin beta-4 chain - Homo sapiens (Human) | 855 | 23 |
| TBB5_HUMAN | Tubulin beta chain - Homo sapiens (Human) | 1082 | 43 |
| TBB6_HUMAN | Tubulin beta-6 chain - Homo sapiens (Human) | 291 | 11 |
| TNKS1_HUMAN | Tankyrase-1 | 40 | 2 |
| WBP4_HUMAN | WW domain-binding protein 4 - Homo sapiens (Human) | 41 | 3 |
