# Supplementary material for: The histone methyltransferase SUV420H2 and Heterochromatin Proteins HP1 interact but show different dynamic behaviours
Source: BMC Cell Biol. 2009 Jun 1;10:41. doi: 10.1186/1471-2121-10-41 (PMC2701926; doi:10.1186/1471-2121-10-41)
Supplement: Additional file 2 — List of peptides identified by TAP with SUV420H2. For each protein, the sequences of the peptides identified by LC-MS/MS in the SUV420H2 tandem affinity purification are listed. [file 1471-2121-10-41-S2.pdf]

List of peptides identified by TAP with SUV420H2

Data represents a total of 485 identified peptides of which 376 are non redundant.
Average mass accuracy is 528ppm; Standard deviation is 520ppm.
Average peptide size is 1558 Da; Standard deviation is 455 Da; Peptide range is 700-3500 Da.

Protein accession	Description	Observed m/z	Peptide Mass (exp. in Da)	Peptide Mass (theo. in Da)	Delta Mass	Peptide Score	Miscle av.	Peptide Sequence	Modifications	Peptide count
5HT7R_HUMAN	5-hydroxytryptamine receptor 7	636,4381	1270,8617	1270,6703	0,1914	14,51	1	KLSAAGMHEALK	Oxidation (M)	1
5HT7R_HUMAN	5-hydroxytryptamine receptor 7	784,419	2350,2353	2351,2379	-1,0026	7,04	3	LQKEVEECANLSRLLKHER	Carbamidomethyl (C)	1
5HT7R_HUMAN	5-hydroxytryptamine receptor 7	517,6117	1549,8133	1547,8824	1,9309	19,49	0	YLGITRPLTYPVR		1
A26CA_HUMAN	ANKRD26-like family C member 1A - Homo sapiens (Human)	741,3866	2221,138	2221,2802	-0,1422	4,26	3	EIAALAPSMMKIRIIAPPKR	Oxidation (M)	1
A26CA_HUMAN	ANKRD26-like family C member 1A - Homo sapiens (Human)	1275,9757	2549,9369	2549,1665	0,7704	75,96	0	LCYVALDFEQEMATAASSSSLEK	Carbamidomethyl (C)	1
A26CA_HUMAN	ANKRD26-like family C member 1A - Homo sapiens (Human)	1284,2669	2566,5192	2565,1614	1,3577	49,5	0	LCYVALDFEQEMATAASSSSLEK	Carbamidomethyl (C); Oxidation (M)	1
A26CA_HUMAN	ANKRD26-like family C member 1A - Homo sapiens (Human)	896,0055	1789,9965	1789,8846	0,1118	79,35	0	SYELPDGQVITIGNER		1
ACTB_HUMAN	Actin, cytoplasmic 1 - Homo sapiens (Human)	600,1538	1198,2931	1197,6982	0,5948	61,02	0	AVFPSIVGRPR		1
ACTB_HUMAN	Actin, cytoplasmic 1 - Homo sapiens (Human)	998,4705	997,4632	997,479	-0,0158	37,88	0	DLTDYLMK		1
ACTB_HUMAN	Actin, cytoplasmic 1 - Homo sapiens (Human)	1014,4752	1013,4679	1013,4739	-0,006	43,49	0	DLTDYLMK	Oxidation (M)	2
ACTB_HUMAN	Actin, cytoplasmic 1 - Homo sapiens (Human)	589,6682	1177,3219	1176,606	0,7159	52,37	0	EITALAPSTMK	Oxidation (M)	1
ACTB_HUMAN	Actin, cytoplasmic 1 - Homo sapiens (Human)	566,8705	1131,7265	1131,5197	0,2068	45,24	0	GYSFTTTAER		2
ACTB_HUMAN	Actin, cytoplasmic 1 - Homo sapiens (Human)	759,2256	1516,4367	1514,7419	1,6948	51,09	0	IWHHTFYNELR		2
ACTB_HUMAN	Actin, cytoplasmic 1 - Homo sapiens (Human)	1180,5657	2359,1168	2358,1526	0,9642	53,52	1	KDLYANTVLSGGTTMYPGIADR	Oxidation (M)	1
ACTB_HUMAN	Actin, cytoplasmic 1 - Homo sapiens (Human)	1284,1577	2566,3008	2565,1614	1,1393	105,26	0	LCYVALDFEQEMATAASSSSLEK	Carbamidomethyl (C); Oxidation (M)	2
ACTB_HUMAN	Actin, cytoplasmic 1 - Homo sapiens (Human)	1275,9757	2549,9369	2549,1665	0,7704	75,96	0	LCYVALDFEQEMATAASSSSLEK	Carbamidomethyl (C)	1
ACTB_HUMAN	Actin, cytoplasmic 1 - Homo sapiens (Human)	506,5678	1516,6816	1515,6954	0,9862	48,12	0	QEYDESGPSIVHR		1
ACTB_HUMAN	Actin, cytoplasmic 1 - Homo sapiens (Human)	896,13	1790,2455	1789,8846	0,3608	86,19	0	SYELPDGQVITIGNER		2
ACTB_HUMAN	Actin, cytoplasmic 1 - Homo sapiens (Human)	977,7029	1953,3912	1953,0571	0,3341	67,79	0	VAPEEHPVLLTEAPLNPK		1
ACTBL_HUMAN	Protein beta-actin-like - Homo sapiens (Human)	998,4705	997,4632	997,479	-0,0158	37,88	0	DLTDYLMK		1
ACTBL_HUMAN	Protein beta-actin-like - Homo sapiens (Human)	1014,4752	1013,4679	1013,4739	-0,006	43,49	0	DLTDYLMK	Oxidation (M)	2
ACTBL_HUMAN	Protein beta-actin-like - Homo sapiens (Human)	580,9648	1159,9151	1158,5305	1,3845	6,45	0	GYNFTTTAER		1
ACTBL_HUMAN	Protein beta-actin-like - Homo sapiens (Human)	896,13	1790,2455	1789,8846	0,3608	86,19	0	SYELPDGQVITIGNER		2
ACTBL_HUMAN	Protein beta-actin-like - Homo sapiens (Human)	977,7029	1953,3912	1953,0571	0,3341	28,45	0	VAPDEHPILLTEAPLNPK		1
ACTC_HUMAN	Actin, alpha cardiac muscle 1 - Homo sapiens (Human)	600,1538	1198,2931	1197,6982	0,5948	61,02	0	AVFPSIVGRPR		1
ACTC_HUMAN	Actin, alpha cardiac muscle 1 - Homo sapiens (Human)	998,4705	997,4632	997,479	-0,0158	37,88	0	DLTDYLMK		1
ACTC_HUMAN	Actin, alpha cardiac muscle 1 - Homo sapiens (Human)	1014,4752	1013,4679	1013,4739	-0,006	43,49	0	DLTDYLMK	Oxidation (M)	2
ACTC_HUMAN	Actin, alpha cardiac muscle 1 - Homo sapiens (Human)	589,6682	1177,3219	1176,606	0,7159	52,37	0	EITALAPSTMK	Oxidation (M)	1
ACTC_HUMAN	Actin, alpha cardiac muscle 1 - Homo sapiens (Human)	1405,7277	2809,4408	2808,2833	1,1574	4,19	1	EKLCYVALDFENEMATAASSSSLEK	Carbamidomethyl (C); Oxidation (M)	1
ACTC_HUMAN	Actin, alpha cardiac muscle 1 - Homo sapiens (Human)	759,2256	1516,4367	1514,7419	1,6948	51,09	0	IWHHTFYNELR		2
ACTC_HUMAN	Actin, alpha cardiac muscle 1 - Homo sapiens (Human)	1275,9757	2549,9369	2551,1458	-1,2089	19,57	0	LCYVALDFENEMATAASSSSLEK	Carbamidomethyl (C); Oxidation (M)	1
ACTC_HUMAN	Actin, alpha cardiac muscle 1 - Homo sapiens (Human)	750,7157	1499,4169	1499,7005	-0,2836	16,73	0	QEYDEAGPSIVHR		2
ACTC_HUMAN	Actin, alpha cardiac muscle 1 - Homo sapiens (Human)	896,13	1790,2455	1789,8846	0,3608	86,19	0	SYELPDGQVITIGNER		2
ACTC_HUMAN	Actin, alpha cardiac muscle 1 - Homo sapiens (Human)	977,7029	1953,3912	1955,0364	-1,6452	11,7	0	VAPEEHPTLLTEAPLNPK		1
ACTC_HUMAN	Actin, alpha cardiac muscle 1 - Homo sapiens (Human)	659,8879	1976,6419	1975,8986	0,7434	20,61	0	YPIEHGIITNWDDMEK	Oxidation (M)	2
ADT2_HUMAN	ADP/ATP translocase 2 - Homo sapiens (Human)	561,5697	1121,1249	1120,5665	0,5583	44,75	0	EQGVLSFWR		1
CALM_HUMAN	Calmodulin - Homo sapiens (Human)	799,2595	1596,5044	1595,7063	0,7981	33,36	1	DTDSEEEIREAFR		1
CALM_HUMAN	Calmodulin - Homo sapiens (Human)	1262,3354	2522,6562	2521,0625	1,5937	42,44	0	EADIDGDGQVNYEEFVQMMTAK	2 Oxidation (M)	1
CALM_HUMAN	Calmodulin - Homo sapiens (Human)	586,215	1755,623	1753,8635	1,7595	24,87	1	VFDKDGNGYISAAELR		1
CBX3_HUMAN	Chromobox protein homolog 3 - Homo sapiens (Human)	1691,9622	3381,9098	3381,4983	0,4115	48,12	0	GFTDADNTWEPEENLDCPELIEAFLNSQK	Carbamidomethyl (C)	1
CBX3_HUMAN	Chromobox protein homolog 3 - Homo sapiens (Human)	857,4803	1712,9461	1711,8525	1,0936	94,72	0	IIGATDSSGELMFLMK		1
CBX3_HUMAN	Chromobox protein homolog 3 - Homo sapiens (Human)	865,1491	1728,2836	1727,8474	0,4362	99,75	0	IIGATDSSGELMFLMK	Oxidation (M)	1
CBX3_HUMAN	Chromobox protein homolog 3 - Homo sapiens (Human)	831,9347	1661,8548	1660,8195	1,0352	100,82	1	KVEEAEPEEFVVEK		7
CBX3_HUMAN	Chromobox protein homolog 3 - Homo sapiens (Human)	767,711	2300,111	2300,2012	-0,0902	7,11	3	KVEEAEPEEFVVEKVLDRR		1
CBX3_HUMAN	Chromobox protein homolog 3 - Homo sapiens (Human)	737,0092	1472,0038	1471,6038	0,4	31,55	0	LTWHSCPEDEAQ	Carbamidomethyl (C)	2
CBX3_HUMAN	Chromobox protein homolog 3 - Homo sapiens (Human)	626,5398	1876,5974	1875,9465	0,6509	17,76	2	SKKVEEAEPEEFVVEK		1
CBX3_HUMAN	Chromobox protein homolog 3 - Homo sapiens (Human)	767,6008	1533,1871	1532,7246	0,4625	62,68	0	VEEAEPEEFVVEK		4
CBX3_HUMAN	Chromobox protein homolog 3 - Homo sapiens (Human)	798,5042	797,4969	797,4323	0,0646	20,96	0	VEYFLK		1

CBX3_HUMAN	Chromobox protein homolog 3 - Homo sapiens (Human)	745,694	1489,3734	1488,746	0,6274	68,85	1	WKDSDEADLVLAK		2
CBX5_HUMAN	Chromobox protein homolog 5 - Homo sapiens (Human)	857,4803	1712,9461	1713,814	-0,8679	29,72	0	IIGATDSCGDLMFLMK		1
CBX5_HUMAN	Chromobox protein homolog 5 - Homo sapiens (Human)	865,1491	1728,2836	1729,8089	-1,5253	8,04	0	IIGATDSCGDLMFLMK	Oxidation (M)	1
CBX5_HUMAN	Chromobox protein homolog 5 - Homo sapiens (Human)	751,6842	1501,3539	1502,7617	-1,4078	16,61	1	WKDTDEADLVLAK		1
CH60_HUMAN	60 kDa heat shock protein, mitochondrial precursor - Homo sapier	886,5869	1771,1593	1770,8458	0,3135	64,92	0	CIPALDSLTPANEDQK	Carbamidomethyl (C)	1
CH60_HUMAN	60 kDa heat shock protein, mitochondrial precursor - Homo sapier	1281,1023	2560,1901	2559,2413	0,9488	12,32	0	LVQDVANNTNEEAGDGTTTATVLAR		1
CH60_HUMAN	60 kDa heat shock protein, mitochondrial precursor - Homo sapier	673,0807	1344,1469	1343,7085	0,4384	56,93	0	TVIIEQSWGSPK		1
CPNS1_HUMAN	Calpain small subunit 1 - Homo sapiens (Human)	870,4406	869,4333	868,4331	1,0003	31,15	0	LGFEEFK		2
CPNS1_HUMAN	Calpain small subunit 1 - Homo sapiens (Human)	571,4807	1140,9469	1140,487	0,4599	51,39	0	TDGFGIDTCR	Carbamidomethyl (C)	3
CPNS1_HUMAN	Calpain small subunit 1 - Homo sapiens (Human)	889,6896	1777,3646	1776,7914	0,5731	85,17	0	THYSNIEANESEEVR		3
DHX9_HUMAN	ATP-dependent RNA helicase A - Homo sapiens (Human)	579,6087	1157,2028	1156,6492	0,5535	45,66	0	VFDPVPVGVTK		1
DNJA1_HUMAN	DnaJ homolog subfamily A member 1 - Homo sapiens (Human)	726,6519	1451,2892	1450,7303	0,5588	78,44	0	QISQAYEVLSDAK		1
DNJA1_HUMAN	DnaJ homolog subfamily A member 1 - Homo sapiens (Human)	697,1755	1392,3364	1391,8137	0,5227	53,18	0	TIVITSHPGQIVK		1
DYH12_HUMAN	Dynein heavy chain 12, axonemal	851,7915	2552,3526	2552,528	-0,1754	13,39	2	LLFDLMPIIWIKPTQKSRIIK		1
DYH12_HUMAN	Dynein heavy chain 12, axonemal	555,4108	1108,8071	1109,4488	-0,6417	27,32	0	WECPFDEK	Carbamidomethyl (C)	1
EF1A1_HUMAN	Elongation factor 1-alpha 1 - Homo sapiens (Human)	658,2588	1314,5031	1313,7343	0,7688	44,89	0	EHALLAYTLGVK		1
EF1A1_HUMAN	Elongation factor 1-alpha 1 - Homo sapiens (Human)	513,4851	1024,9556	1024,603	0,3526	65,94	0	IGGIGTVPVGR		1
EF1A1_HUMAN	Elongation factor 1-alpha 1 - Homo sapiens (Human)	702,9696	1403,9247	1403,7197	0,2049	57,29	0	YYVTIIDAPGHR		2
EFTU_HUMAN	Elongation factor Tu, mitochondrial precursor - Homo sapiens (Hu	593,4229	1184,8313	1184,6149	0,2163	112,65	0	AEAGDNLGALVR		1
EFTU_HUMAN	Elongation factor Tu, mitochondrial precursor - Homo sapiens (Hu	838,1967	1674,3789	1672,8533	1,5256	75,62	0	GITINAAHVEYSTAAR		1
EFTU_HUMAN	Elongation factor Tu, mitochondrial precursor - Homo sapiens (Hu	747,3067	1492,5988	1491,6841	0,9147	31,7	1	KYEEIDNAPEER		1
EFTU_HUMAN	Elongation factor Tu, mitochondrial precursor - Homo sapiens (Hu	742,7276	1483,4406	1482,8195	0,6211	62,99	0	QIGVEHVVVYVNK		1
GRP78_HUMAN	78 kDa glucose-regulated protein precursor - Homo sapiens (Hum	617,7313	1233,4481	1232,6183	0,8298	91,35	0	DAGTIAGLNVMR	Oxidation (M)	1
GRP78_HUMAN	78 kDa glucose-regulated protein precursor - Homo sapiens (Hum	1088,902	2175,7894	2174,9855	0,8039	17,37	1	LYGSAGPPPTGEEDTAEKDEL		1
GRP78_HUMAN	78 kDa glucose-regulated protein precursor - Homo sapiens (Hum	839,594	1677,1734	1676,8006	0,3728	72,82	0	NQLTSNPENTVFDAK		1
GRP78_HUMAN	78 kDa glucose-regulated protein precursor - Homo sapiens (Hum	919,3602	1836,7059	1835,9265	0,7794	64,73	0	SQIFSTASDNQPTVTIK		1
GRP78_HUMAN	78 kDa glucose-regulated protein precursor - Homo sapiens (Hum	716,0039	1429,9933	1429,6838	0,3095	68,57	0	TWNDPSVQQDIK		1
GRP78_HUMAN	78 kDa glucose-regulated protein precursor - Homo sapiens (Hum	944,7859	1887,5573	1886,9639	0,5934	88,67	0	VTHAVVTVPAYFNDAQR		1
GTF2I_HUMAN	General transcription factor II-I - Homo sapiens (Human)	624,5535	1247,0924	1246,6921	0,4003	89,17	0	FAQALGLTEAVK		1
H2A1D_HUMAN	Histone H2A type 1-D - Homo sapiens (Human)	637,0766	1272,1387	1271,6721	0,4666	63,97	1	NDEELNKLLGK		2
H2A1D_HUMAN	Histone H2A type 1-D - Homo sapiens (Human)	966,4126	1930,8107	1930,1615	0,6491	70,1	0	VTIAQGGVLPNIQAVLLPK		2
H2B1C_HUMAN	Histone H2B type 1-C/E/F/G/I - Homo sapiens (Human)	888,8372	1775,6599	1774,8018	0,858	79,12	0	AMGIMNSFVNDIFER	2 Oxidation (M)	1
H2B1C_HUMAN	Histone H2B type 1-C/E/F/G/I - Homo sapiens (Human)	1137,5757	1136,5684	1136,539	0,0295	25,67	0	ESYSVYVYK		1
H2B1C_HUMAN	Histone H2B type 1-C/E/F/G/I - Homo sapiens (Human)	953,6213	952,614	952,5957	0,0183	18,62	0	LLLPGELAK		1
H31T_HUMAN	Histone H3.1t - Homo sapiens (Human)	850,4722	849,4649	849,4232	0,0417	54,14	0	EIAQDFK		1
H31T_HUMAN	Histone H3.1t - Homo sapiens (Human)	668,6259	1335,2373	1334,683	0,5543	46,91	1	EIAQDFKTDLR		2
H31T_HUMAN	Histone H3.1t - Homo sapiens (Human)	517,2225	1548,6458	1549,8953	-1,2495	10,87	1	KPHRYRPGTVALR		1
H31T_HUMAN	Histone H3.1t - Homo sapiens (Human)	516,9509	1031,8873	1031,5876	0,2997	24,91	0	YRPGTVALR		4
H4_HUMAN	Histone H4 - Homo sapiens (Human)	567,8659	1133,7173	1133,5353	0,182	43,03	0	DAVTYTEHAK		1
H4_HUMAN	Histone H4 - Homo sapiens (Human)	663,4572	1324,8998	1324,7463	0,1535	49,5	0	DNIQGITKPAIR		2
H4_HUMAN	Histone H4 - Homo sapiens (Human)	591,2062	1180,3978	1179,6135	0,7842	54,35	0	ISGLIYEETR		1
H4_HUMAN	Histone H4 - Homo sapiens (Human)	714,6715	713,6642	713,3385	0,3258	43,29	0	TLYGFGG		2
H4_HUMAN	Histone H4 - Homo sapiens (Human)	989,5863	988,579	988,5706	0,0085	23,33	0	VFLENVIR		1
HNRL2_HUMAN	Heterogeneous nuclear ribonucleoprotein U-like protein 2 - Homo	915,0945	1828,1745	1826,837	1,3375	66,65	0	NFILDQCNVYNSGQR	Carbamidomethyl (C)	1
HNRPU_HUMAN	Heterogeneous nuclear ribonucleoprotein U - Homo sapiens (Hum	824,9541	1647,8936	1646,8376	1,056	104,71	0	NFILDQTNVSAAAQR		1
HNRPU_HUMAN	Heterogeneous nuclear ribonucleoprotein U - Homo sapiens (Hum	525,4044	1048,7943	1047,5349	1,2594	36,67	0	NGQDLGVAFK		1
HS90B_HUMAN	Heat shock protein HSP 90-beta - Homo sapiens (Human)	924,7977	1847,5809	1846,7897	0,7912	69,45	0	NPDDITQEEYGEFYK		2
HSP7C_HUMAN	Heat shock cognate 71 kDa protein - Homo sapiens (Human)	600,7439	1199,4733	1198,667	0,8063	71,94	0	DAGTIAGLNVLR		1
HSP7C_HUMAN	Heat shock cognate 71 kDa protein - Homo sapiens (Human)	626,876	1251,7374	1251,6533	0,0841	75,3	1	MKEIAEAYLGK		1
HSP7C_HUMAN	Heat shock cognate 71 kDa protein - Homo sapiens (Human)	825,866	1649,7175	1648,7879	0,9296	81,79	0	NQVAMNPTNTVFDAK		1
HSP7C_HUMAN	Heat shock cognate 71 kDa protein - Homo sapiens (Human)	808,9172	1615,8198	1615,7803	0,0394	79,47	0	SFYPEEVSSMVLTK		1
HSP7C_HUMAN	Heat shock cognate 71 kDa protein - Homo sapiens (Human)	741,7506	1481,4867	1480,7998	0,6869	55,73	0	SQIHDIVLVGGSTR		1
HSP7C_HUMAN	Heat shock cognate 71 kDa protein - Homo sapiens (Human)	744,8338	1487,6531	1486,694	0,9591	55,14	0	TTPSYVAFTDTER		1
IQGA1_HUMAN	Ras GTPase-activating-like protein IQGAP1 - Homo sapiens (Hum	863,0733	1724,132	1723,8377	0,2943	74,66	0	EEIQSSISGVTAAYNR		1
K1967_HUMAN	Protein KIAA1967 - Homo sapiens (Human)	589,5165	1177,0184	1176,5808	0,4375	23,17	0	TLAAEMQELR	Oxidation (M)	1
K1967_HUMAN	Protein KIAA1967 - Homo sapiens (Human)	641,0996	1280,1846	1279,6343	0,5503	59,82	0	VVTQNICQYR	Carbamidomethyl (C)	1
K6PP_HUMAN	6-phosphofructokinase type C - Homo sapiens (Human)	902,5745	1803,1344	1802,8945	0,2399	87,27	0	AIGVLTSGGDAQGMNAAVR	Oxidation (M)	1

K6PP_HUMAN	6-phosphofructokinase type C - Homo sapiens (Human)	707,1185	1412,2225	1411,6943	0,5282	61,62	0	DLQSNVEHLTEK		1
K6PP_HUMAN	6-phosphofructokinase type C - Homo sapiens (Human)	1104,4078	3310,2016	3309,5726	0,629	5,41	3	LPLMECVQMTQDVQKAMDERRFQDAVR	Carbamidomethyl (C); Oxidation (M)	1
K6PP_HUMAN	6-phosphofructokinase type C - Homo sapiens (Human)	1339,4993	2676,984	2676,1173	0,8666	70,59	0	NESCSENYTTDFIYQLYSEEGK	Carbamidomethyl (C)	1
KU70_HUMAN	ATP-dependent DNA helicase 2 subunit 1 - Homo sapiens (Huma	852,7406	1703,4666	1702,8063	0,6602	50,81	0	SDSFENPVLQQHFR		1
KU86_HUMAN	ATP-dependent DNA helicase 2 subunit 2 - Homo sapiens (Huma	537,4264	1072,8383	1072,624	0,2142	71,06	0	LTIGSNLSIR		1
LHX1_HUMAN	LIM/homeobox protein Lhx1 - Homo sapiens (Human)	624,1989	1246,3832	1245,6975	0,6856	32,42	2	RMKQLSALGAR	Oxidation (M)	1
LHX1_HUMAN	LIM/homeobox protein Lhx1 - Homo sapiens (Human)	569,7003	1706,0792	1705,6907	0,3885	13,25	0	VFHLNCFTCMMCNK	Oxidation (M)	1
MATR3_HUMAN	Matrin-3 - Homo sapiens (Human)	897,5335	1793,0525	1791,8751	1,1774	74,15	0	GDADQASNILASFGLSAR		1
MATR3_HUMAN	Matrin-3 - Homo sapiens (Human)	663,1052	1324,1958	1323,6644	0,5314	10,43	0	GNLGAGNGNLQGPR		1
MATR3_HUMAN	Matrin-3 - Homo sapiens (Human)	680,0995	2037,2765	2036,0691	1,2074	38,19	0	VIHLSNLPHSGYSDSAVLK		2
MCM7_HUMAN	DNA replication licensing factor MCM7 - Homo sapiens (Human)	515,3344	1028,6542	1028,5978	0,0563	57,93	0	AGILTTLNAR		1
MCM7_HUMAN	DNA replication licensing factor MCM7 - Homo sapiens (Human)	636,4381	1270,8617	1270,5717	0,29	43,35	0	QIAEEDFYEK		1
MCM7_HUMAN	DNA replication licensing factor MCM7 - Homo sapiens (Human)	737,7227	1473,4308	1472,81	0,6208	46,64	0	TQRPADVIFATVR		1
MFAP4_HUMAN	Microfibril-associated glycoprotein 4 precursor - Homo sapiens (Hu	1051,72	2101,4254	2101,0666	0,3588	28,54	0	ADGEYWLGLQNMHLLTLK		1
MFAP4_HUMAN	Microfibril-associated glycoprotein 4 precursor - Homo sapiens (Hu	877,3724	876,3651	876,4381	-0,073	17,64	0	GFYYSLK		1
MFAP4_HUMAN	Microfibril-associated glycoprotein 4 precursor - Homo sapiens (Hu	517,3956	1032,7767	1032,5392	0,2374	28,75	1	GFYYSLKR		1
MPCP_HUMAN	Phosphate carrier protein, mitochondrial precursor - Homo sapiens	681,7123	1361,41	1360,7099	0,7001	43,96	0	IQTQPGYANTLR		1
MYH14_HUMAN	Myosin-14	653,5874	1305,1603	1304,6612	0,4991	67,87	0	EQADFALEALAK		1
MYH9_HUMAN	Myosin-9 - Homo sapiens (Human)	597,8327	1193,6508	1192,6088	1,042	52,07	0	ALELDSNLYR		1
MYH9_HUMAN	Myosin-9 - Homo sapiens (Human)	523,355	1567,0432	1566,6879	0,3554	10,31	2	DCMRELDDTRASR		1
MYH9_HUMAN	Myosin-9 - Homo sapiens (Human)	653,5874	1305,1603	1304,6612	0,4991	67,87	0	EQADFAIEALAK		1
MYH9_HUMAN	Myosin-9 - Homo sapiens (Human)	827,6762	1653,3378	1652,7781	0,5597	100,85	0	IAEFTTNLTEEEEK		1
MYH9_HUMAN	Myosin-9 - Homo sapiens (Human)	935,4059	1868,7973	1867,9051	0,8922	74,03	1	IAEFTTNLTEEEEKSK		1
MYH9_HUMAN	Myosin-9 - Homo sapiens (Human)	766,2333	1530,452	1529,7573	0,6947	88,43	0	IAQLEEQLDNETK		2
MYH9_HUMAN	Myosin-9 - Homo sapiens (Human)	973,7279	1945,4413	1945,0004	0,4409	85,06	0	LQVELDNVTGLLSQSDSK		1
MYH9_HUMAN	Myosin-9 - Homo sapiens (Human)	837,3958	1672,777	1671,8369	0,9401	73,77	0	NFINNPLAQADWAAK		1
MYH9_HUMAN	Myosin-9 - Homo sapiens (Human)	603,5266	1205,0387	1204,6339	0,4047	63,44	0	TDLLLEPYNK		1
MYLK2_HUMAN	Myosin light chain kinase 2, skeletal/cardiac muscle - Homo sapie	1141,1022	3420,2849	3420,7307	-0,4458	6,18	2	MATENGAVELGIQNPSTDKAPKGPTGERPLA/	Oxidation (M)	1
MYLK2_HUMAN	Myosin light chain kinase 2, skeletal/cardiac muscle - Homo sapie	531,9875	1061,9605	1061,5618	0,3987	65,13	0	NFIAVSAANR		5
OFD1_HUMAN	Oral-facial-digital syndrome 1 protein	738,6143	1475,2141	1474,8792	0,3348	24,82	3	KMIEESLKIKIK	Oxidation (M)	1
OFD1_HUMAN	Oral-facial-digital syndrome 1 protein	565,9241	1129,8336	1129,5437	0,2898	17,94	0	MPLPSPTESR	Oxidation (M)	1
PP1A_HUMAN	Serine/threonine-protein phosphatase PP1-alpha catalytic subunit	820,9157	1639,8169	1638,7678	1,0491	58,14	0	AHQVVEDGYEFFAK		1
PYR1_HUMAN	CAD protein [Includes: Glutamine-dependent carbamoyl-phosphat	612,9771	1223,9396	1223,655	0,2846	60,07	0	ATGYPLAYVAAK		1
PYR1_HUMAN	CAD protein [Includes: Glutamine-dependent carbamoyl-phosphat	501,9514	1001,8883	1001,5579	0,3303	37,94	0	ILALDCGLK	Carbamidomethyl (C)	1
PYR1_HUMAN	CAD protein [Includes: Glutamine-dependent carbamoyl-phosphat	603,5266	1205,0387	1206,6795	-1,6408	16,56	1	MALLATVLGRF	Oxidation (M)	1
PYR1_HUMAN	CAD protein [Includes: Glutamine-dependent carbamoyl-phosphat	780,1218	1558,2291	1557,7634	0,4656	100,23	0	VLGTSPEAIDSAENR		1
RL11_HUMAN	60S ribosomal protein L11 - Homo sapiens (Human)	774,2217	1546,4288	1545,8403	0,5885	81,51	0	VLEQLTGQTPVFSK		1
RL12_HUMAN	60S ribosomal protein L12 - Homo sapiens (Human)	838,8924	1675,7702	1674,7995	0,9706	68,39	0	EILGTAQSVGCNVDGR	Carbamidomethyl (C)	1
RL23_HUMAN	60S ribosomal protein L23 - Homo sapiens (Human)	746,3539	1490,6933	1490,7473	-0,054	84,26	0	LPAAGVGDMVMATVK	2 Oxidation (M)	1
RL23A_HUMAN	60S ribosomal protein L23a - Homo sapiens (Human)	703,0634	1404,1122	1403,6932	0,4189	79,59	0	LAPDYDALDVANK		1
RL27A_HUMAN	60S ribosomal protein L27a - Homo sapiens (Human)	932,3718	931,3645	931,4797	-0,1152	8,73	0	SVGGACVLVA	Carbamidomethyl (C)	1
RL27A_HUMAN	60S ribosomal protein L27a - Homo sapiens (Human)	556,5784	1111,1422	1110,6397	0,5025	69,75	0	TGAAPIIDVVR		1
RL30_HUMAN	60S ribosomal protein L30 - Homo sapiens (Human)	879,6231	1757,2316	1756,9029	0,3286	58,16	0	VCTLAIIDPGDSDIIR	Carbamidomethyl (C)	1
RLA0_HUMAN	60S acidic ribosomal protein P0 - Homo sapiens (Human)	633,9716	1265,9286	1265,6074	0,3211	66,12	0	CFIVGADNVGSK	Carbamidomethyl (C)	1
RLA0_HUMAN	60S acidic ribosomal protein P0 - Homo sapiens (Human)	611,7721	1221,5297	1220,6149	0,9148	32,25	0	GHLENNPALEK		1
RLA0_HUMAN	60S acidic ribosomal protein P0 - Homo sapiens (Human)	560,7328	1679,1764	1678,9044	0,272	5,13	3	GKAVVLMGKNTMMRK	Oxidation (M)	1
RLA1_HUMAN	60S acidic ribosomal protein P1 - Homo sapiens (Human)	852,7785	1703,5425	1701,8879	1,6546	58,32	0	AAGVNVEPFWPGLFAK		1
RS11_HUMAN	40S ribosomal protein S11 - Homo sapiens (Human)	576,0567	1150,0988	1149,5601	0,5387	50,36	0	CPFTGNVSIR	Carbamidomethyl (C)	1
RS11_HUMAN	40S ribosomal protein S11 - Homo sapiens (Human)	993,9125	1985,8104	1985,0252	0,7852	32,17	0	DVQIGDIVTVGECRPLSK	Carbamidomethyl (C)	1
RS13_HUMAN	40S ribosomal protein S13 - Homo sapiens (Human)	624,6682	1247,3218	1246,6782	0,6436	13,48	1	GLSQSALPYRR		1
RS13_HUMAN	40S ribosomal protein S13 - Homo sapiens (Human)	720,082	1438,1494	1437,7351	0,4143	63,65	1	LTSDDVKEQIYK		1
RS15A_HUMAN	40S ribosomal protein S15a - Homo sapiens (Human)	743,3909	742,3836	742,4054	-0,0218	12,95	0	ILGFFF		1
RS15A_HUMAN	40S ribosomal protein S15a - Homo sapiens (Human)	564,4048	1126,7951	1126,5883	0,2068	37,71	0	WQNNLLPSR		1
RS17_HUMAN	40S ribosomal protein S17 - Homo sapiens (Human)	630,1209	1258,2273	1257,6638	0,5634	65,14	0	VCEEIAIIPSK	Carbamidomethyl (C)	1
RS18_HUMAN	40S ribosomal protein S18 - Homo sapiens (Human)	624,6682	1247,3218	1246,5677	0,7541	44,7	0	AGELTEDEVER		1
RS18_HUMAN	40S ribosomal protein S18 - Homo sapiens (Human)	531,0832	1060,1519	1059,5502	0,6017	37,55	0	IPDWFLNR		1
								LITPAVVSER		

RS3_HUMAN	40S ribosomal protein S3 - Homo sapiens (Human)	547,0076	1092,0007	1091,5611	0,4396	44,72	0	AELNEFLTR		1
RS3_HUMAN	40S ribosomal protein S3 - Homo sapiens (Human)	736,0575	1470,1004	1469,7613	0,3391	55,46	0	DEILPTTPISEQK		1
RS3_HUMAN	40S ribosomal protein S3 - Homo sapiens (Human)	712,6112	1423,2079	1422,6627	0,5452	62,16	0	ELAEDGYSGVEVR		1
RS3_HUMAN	40S ribosomal protein S3 - Homo sapiens (Human)	787,8114	1573,6083	1572,797	0,8113	46,46	0	GGKPEPPAMPQPVPTA		1
RS3_HUMAN	40S ribosomal protein S3 - Homo sapiens (Human)	795,8562	1589,6979	1588,7919	0,9059	41,3	0	GGKPEPPAMPQPVPTA	Oxidation (M)	1
RS3_HUMAN	40S ribosomal protein S3 - Homo sapiens (Human)	645,2476	1288,4806	1287,6605	0,8201	57,52	0	GLCAIAQAESLR	Carbamidomethyl (C)	1
RS3_HUMAN	40S ribosomal protein S3 - Homo sapiens (Human)	515,3828	1028,7511	1028,623	0,1281	68,99	0	TEIIILATR		1
RS3A_HUMAN	40S ribosomal protein S3a - Homo sapiens (Human)	664,5728	1327,131	1326,7395	0,3915	49,36	1	LIPDSIGKDIEK		1
RS4Y1_HUMAN	40S ribosomal protein S4, Y isoform 1 - Homo sapiens (Human)	608,4828	1214,9511	1214,652	0,2991	59,02	0	GIPHLVTHDAR		1
RS5_HUMAN	40S ribosomal protein S5 - Homo sapiens (Human)	816,8069	1631,5993	1630,8236	0,7757	62,05	0	TIAECLADELINAAK	Carbamidomethyl (C)	1
S10A2_HUMAN	Protein S100-A2 - Homo sapiens (Human)	503,3832	1004,5179	0,2339	1004,752	38,39	0	ELPSFVGEK		1
S10A2_HUMAN	Protein S100-A2 - Homo sapiens (Human)	952,703	1902,9938	0,3976	1903,392	47,66	2	ELPSFVGEKVDEEGLKK		2
SFPQ_HUMAN	Splicing factor, proline- and glutamine-rich - Homo sapiens (Huma	627,0319	1252,0492	1251,6135	0,4356	74,47	0	YGEPGEVFINK		1
SP16H_HUMAN	FACT complex subunit SPT16 - Homo sapiens (Human)	713,3916	1424,7687	1424,6936	0,0751	67,62	0	YTEGVQSLNWTK		1
SPG7_HUMAN	Paraplegin - Homo sapiens (Human)	737,2205	1472,4264	1470,7678	1,6586	40,19	1	LRAAEDELNIEAK		1
SPG7_HUMAN	Paraplegin - Homo sapiens (Human)	1104,8129	3311,4167	3312,6013	-1,1846	3,74	3	MCMALGGRASEALSFNEVTSGAQDDLRKVTR		1
SPTA2_HUMAN	Spectrin alpha chain, brain - Homo sapiens (Human)	697,6912	1393,3678	1392,6997	0,668	68,19	0	LGESQTLQQFSR		1
SPTA2_HUMAN	Spectrin alpha chain, brain - Homo sapiens (Human)	537,4167	1072,8189	1072,5513	0,2676	34,14	0	SLQQLAEER		1
SSB_HUMAN	Single-stranded DNA-binding protein, mitochondrial precursor - Hc	806,9005	1611,7865	1610,7424	1,0441	67,83	0	SGDSEVYQLGDVSQK		1
**SUV42_HUMAN**	**Histone-lysine N-methyltransferase SUV420H2 - Homo sapien**	**855,1813**	**1708,348**	**1707,8654**	**0,4825**	**67,27**	**0**	**AFLPESGFTILPCTR**	**Carbamidomethyl (C)**	**3**
**SUV42_HUMAN**	**Histone-lysine N-methyltransferase SUV420H2 - Homo sapien**	**746,0521**	**1490,0896**	**1489,6507**	**0,4388**	**50,9**	**0**	**AGENDFSIMYSTR**		**1**
**SUV42_HUMAN**	**Histone-lysine N-methyltransferase SUV420H2 - Homo sapien**	**754,2528**	**1506,4911**	**1505,6456**	**0,8454**	**89,37**	**0**	**AGENDFSIMYSTR**	**Oxidation (M)**	**2**
**SUV42_HUMAN**	**Histone-lysine N-methyltransferase SUV420H2 - Homo sapien**	**523,4085**	**1044,8024**	**1044,5716**	**0,2307**	**48,44**	**0**	**ALTLGGWTAR**		**3**
**SUV42_HUMAN**	**Histone-lysine N-methyltransferase SUV420H2 - Homo sapien**	**668,0097**	**1334,0048**	**1333,5907**	**0,414**	**55,88**	**0**	**DPFCAACQPLR**	**2 Carbamidomethyl (C)**	**1**
**SUV42_HUMAN**	**Histone-lysine N-methyltransferase SUV420H2 - Homo sapien**	**902,4431**	**901,4358**	**901,4505**	**-0,0147**	**40,36**	**0**	**EADEGLLR**		**1**
**SUV42_HUMAN**	**Histone-lysine N-methyltransferase SUV420H2 - Homo sapien**	**617,1085**	**1232,2024**	**1231,6924**	**0,5099**	**32,33**	**0**	**EPALPPRPLDK**		**1**
**SUV42_HUMAN**	**Histone-lysine N-methyltransferase SUV420H2 - Homo sapien**	**625,0119**	**1248,0093**	**1247,5969**	**0,4124**	**26,81**	**0**	**FVPADGNAACVK**	**Carbamidomethyl (C)**	**1**
**SUV42_HUMAN**	**Histone-lysine N-methyltransferase SUV420H2 - Homo sapien**	**693,7311**	**1385,4476**	**1384,7748**	**0,6728**	**81,97**	**0**	**LELLVGCIAELR**	**Carbamidomethyl (C)**	**1**
**SUV42_HUMAN**	**Histone-lysine N-methyltransferase SUV420H2 - Homo sapien**	**842,6008**	**1683,1871**	**1683,9532**	**-0,7662**	**46,4**	**1**	**LRGEALVALGQPPHAR**		**1**
**SUV42_HUMAN**	**Histone-lysine N-methyltransferase SUV420H2 - Homo sapien**	**870,35**	**1738,6854**	**1737,8785**	**0,8069**	**50,56**	**0**	**LVVSHGSIDLDVGGEEL**		**1**
**SUV42_HUMAN**	**Histone-lysine N-methyltransferase SUV420H2 - Homo sapien**	**633,697**	**1265,3795**	**1264,7074**	**0,6721**	**23,75**	**1**	**MNVSPVPPLRR**		**1**
**SUV42_HUMAN**	**Histone-lysine N-methyltransferase SUV420H2 - Homo sapien**	**561,9517**	**1121,8889**	**1121,6346**	**0,2543**	**25,42**	**1**	**RYGLPYVVR**		**1**
**SUV42_HUMAN**	**Histone-lysine N-methyltransferase SUV420H2 - Homo sapien**	**810,0672**	**2427,1797**	**2426,1623**	**1,0174**	**27,64**	**0**	**SAQLWLGPAAFINHDCKPNCK**	**2 Carbamidomethyl (C)**	**1**
**SUV42_HUMAN**	**Histone-lysine N-methyltransferase SUV420H2 - Homo sapien**	**966,5652**	**965,5579**	**965,5334**	**0,0245**	**11,52**	**0**	**YGLPYVVR**		**1**
TBA1A_HUMAN	Tubulin alpha-1A chain - Homo sapiens (Human)	860,0285	1718,0425	1717,8747	0,1678	60,84	0	NLDIERPTYTNLNR		2
TBA1B_HUMAN	Tubulin alpha-1B chain - Homo sapiens (Human)	933,4362	1864,8578	1863,8971	0,9607	93,74	0	AVCMLSNTTAIAEAWAR	Carbamidomethyl (C)	1
TBA1B_HUMAN	Tubulin alpha-1B chain - Homo sapiens (Human)	851,5945	1701,1745	1700,8985	0,276	48,41	0	AVFVDLEPTVIDEVR		1
TBA1B_HUMAN	Tubulin alpha-1B chain - Homo sapiens (Human)	917,8506	2750,53	2749,284	1,2461	32,74	0	AYHEQLSVAEITNACFEPANQMVK	Carbamidomethyl (C)	1
TBA1B_HUMAN	Tubulin alpha-1B chain - Homo sapiens (Human)	923,0939	2766,2599	2765,2789	0,9811	30,74	0	AYHEQLSVAEITNACFEPANQMVK	Carbamidomethyl (C); Oxidation (M)	1
TBA1B_HUMAN	Tubulin alpha-1B chain - Homo sapiens (Human)	508,5311	1015,0476	1014,5709	0,4766	57,85	0	DVNAAIATIK		1
TBA1B_HUMAN	Tubulin alpha-1B chain - Homo sapiens (Human)	1619,1188	3236,223	3235,3034	0,9196	65,43	1	EDMAALEKDYEEVGVDSVEGEGEEEGEEY		1
TBA1B_HUMAN	Tubulin alpha-1B chain - Homo sapiens (Human)	879,2761	1756,5377	1755,9559	0,5817	60,28	0	IHFPLATYAPVISAEK		1
TBA1B_HUMAN	Tubulin alpha-1B chain - Homo sapiens (Human)	699,0307	1396,0468	1395,6857	0,3611	52,01	1	LDHKFDLMYAK	Oxidation (M)	1
TBA1B_HUMAN	Tubulin alpha-1B chain - Homo sapiens (Human)	744,7038	1487,393	1486,8719	0,5211	56,85	0	LISQIVSSITASLR		1
TBA1B_HUMAN	Tubulin alpha-1B chain - Homo sapiens (Human)	860,1944	1718,3742	1717,8747	0,4995	30,84	0	NLDIERPTYTNLNR		1
TBA1B_HUMAN	Tubulin alpha-1B chain - Homo sapiens (Human)	938,3579	1874,7013	1873,9758	0,7255	26,79	1	RNLDIERPTYTNLNR		2
TBA1B_HUMAN	Tubulin alpha-1B chain - Homo sapiens (Human)	793,2326	1584,4507	1583,7443	0,7064	68,85	0	SIQFVDWCPTGFK	Carbamidomethyl (C)	1
TBA1B_HUMAN	Tubulin alpha-1B chain - Homo sapiens (Human)	1826,2119	1825,2046	1823,9782	1,2265	37,22	0	VGINYQPPTVVPGGDLAK		1
TBA1B_HUMAN	Tubulin alpha-1B chain - Homo sapiens (Human)	625,6317	1249,2488	1248,5453	0,7034	29,29	0	YMACCLLYR	2 Carbamidomethyl (C)	1
TBA1B_HUMAN	Tubulin alpha-1B chain - Homo sapiens (Human)	633,4175	1264,8204	1264,5403	0,2801	50,39	0	YMACCLLYR	2 Carbamidomethyl (C); Oxidation (M)	1
TBA1C_HUMAN	Tubulin alpha-1C chain - Homo sapiens (Human)	926,1551	1850,2957	1849,8815	0,4142	91,52	0	AVCMLSNTTAVAEAWAR	Carbamidomethyl (C)	1
TBA1C_HUMAN	Tubulin alpha-1C chain - Homo sapiens (Human)	933,4362	1864,8578	1865,8764	-1,0186	7,5	0	AVCMLSNTTAVAEAWAR	Carbamidomethyl (C); Oxidation (M)	1
TBA1C_HUMAN	Tubulin alpha-1C chain - Homo sapiens (Human)	851,5945	1701,1745	1700,8985	0,276	48,41	0	AVFVDLEPTVIDEVR		1
TBA1C_HUMAN	Tubulin alpha-1C chain - Homo sapiens (Human)	508,5311	1015,0476	1014,5709	0,4766	57,85	0	DVNAAIATIK		2
TBA1C_HUMAN	Tubulin alpha-1C chain - Homo sapiens (Human)	879,2761	1756,5377	1755,9559	0,5817	60,28	0	IHFPLATYAPVISAEK		1
TBA1C_HUMAN	Tubulin alpha-1C chain - Homo sapiens (Human)	699,0307	1396,0468	1395,6857	0,3611	52,01	1	LDHKFDLMYAK	Oxidation (M)	1
								LISQIVSSITASLR		

TBA1C_HUMAN	Tubulin alpha-1C chain - Homo sapiens (Human)	860,3447	1718,6749	1717,8747	0,8002	49,63	0	NLDIERPTYTNLNR		5
TBA1C_HUMAN	Tubulin alpha-1C chain - Homo sapiens (Human)	938,3579	1874,7013	1873,9758	0,7255	26,79	1	RNLDIERPTYTNLNR		2
TBA1C_HUMAN	Tubulin alpha-1C chain - Homo sapiens (Human)	913,0072	1823,9999	1823,9782	0,0217	62,92	0	VGINYQPPTVVPGGDLAK		6
TBA1C_HUMAN	Tubulin alpha-1C chain - Homo sapiens (Human)	625,6317	1249,2488	1248,5453	0,7034	29,29	0	YMACCLLYR	2 Carbamidomethyl (C)	1
TBA1C_HUMAN	Tubulin alpha-1C chain - Homo sapiens (Human)	633,4175	1264,8204	1264,5403	0,2801	50,39	0	YMACCLLYR	2 Carbamidomethyl (C); Oxidation (M)	1
TBA4A_HUMAN	Tubulin alpha-4A chain - Homo sapiens (Human)	933,4362	1864,8578	1863,8971	0,9607	93,74	0	AVCMLSNTTAIAEAWAR	Carbamidomethyl (C)	1
TBA4A_HUMAN	Tubulin alpha-4A chain - Homo sapiens (Human)	858,6771	1715,3396	1714,9142	0,4254	72,59	0	AVFVDLEPTVIDEIR		1
TBA4A_HUMAN	Tubulin alpha-4A chain - Homo sapiens (Human)	917,8506	2750,53	2749,284	1,2461	32,74	0	AYHEQLSVAEITNACFEPANQMVK	Carbamidomethyl (C)	1
TBA4A_HUMAN	Tubulin alpha-4A chain - Homo sapiens (Human)	923,0939	2766,2599	2765,2789	0,9811	30,74	0	AYHEQLSVAEITNACFEPANQMVK	Carbamidomethyl (C); Oxidation (M)	1
TBA4A_HUMAN	Tubulin alpha-4A chain - Homo sapiens (Human)	985,5384	984,5311	984,5604	-0,0292	38,73	0	DVNAAIAAIK		1
TBA4A_HUMAN	Tubulin alpha-4A chain - Homo sapiens (Human)	535,3499	1068,6852	1068,5815	0,1037	37,96	0	EIIDPVLDR		1
TBA4A_HUMAN	Tubulin alpha-4A chain - Homo sapiens (Human)	879,2761	1756,5377	1755,9559	0,5817	60,28	0	IHFPLATYAPVISAEK		1
TBA4A_HUMAN	Tubulin alpha-4A chain - Homo sapiens (Human)	699,0307	1396,0468	1395,6857	0,3611	52,01	1	LDHKFDLMYAK	Oxidation (M)	1
TBA4A_HUMAN	Tubulin alpha-4A chain - Homo sapiens (Human)	744,7038	1487,393	1486,8719	0,5211	56,85	0	LISQIVSSITASLR		1
TBA4A_HUMAN	Tubulin alpha-4A chain - Homo sapiens (Human)	860,1944	1718,3742	1717,8747	0,4995	30,84	0	NLDIERPTYTNLNR		1
TBA4A_HUMAN	Tubulin alpha-4A chain - Homo sapiens (Human)	938,3579	1874,7013	1873,9758	0,7255	26,79	1	RNLDIERPTYTNLNR		2
TBA4A_HUMAN	Tubulin alpha-4A chain - Homo sapiens (Human)	793,2326	1584,4507	1583,7443	0,7064	68,85	0	SIQFVDWCPTGFK	Carbamidomethyl (C)	1
TBA4A_HUMAN	Tubulin alpha-4A chain - Homo sapiens (Human)	1826,2119	1825,2046	1823,9782	1,2265	37,22	0	VGINYQPPTVVPGGDLAK		1
TBA4A_HUMAN	Tubulin alpha-4A chain - Homo sapiens (Human)	625,6317	1249,2488	1248,5453	0,7034	29,29	0	YMACCLLYR	2 Carbamidomethyl (C)	1
TBA4A_HUMAN	Tubulin alpha-4A chain - Homo sapiens (Human)	633,4175	1264,8204	1264,5403	0,2801	50,39	0	YMACCLLYR	2 Carbamidomethyl (C); Oxidation (M)	1
TBA8_HUMAN	Tubulin alpha-8 chain - Homo sapiens (Human)	933,4362	1864,8578	1863,8971	0,9607	93,74	0	AVCMLSNTTAIAEAWAR	Carbamidomethyl (C)	1
TBA8_HUMAN	Tubulin alpha-8 chain - Homo sapiens (Human)	851,5945	1701,1745	1700,8655	0,309	10,9	0	AVMIDLEPTVVDEVR	Oxidation (M)	1
TBA8_HUMAN	Tubulin alpha-8 chain - Homo sapiens (Human)	699,0307	1396,0468	1395,6857	0,3611	52,01	1	LDHKFDLMYAK	Oxidation (M)	1
TBA8_HUMAN	Tubulin alpha-8 chain - Homo sapiens (Human)	744,7038	1487,393	1486,8719	0,5211	56,85	0	LISQIVSSITASLR		1
TBA8_HUMAN	Tubulin alpha-8 chain - Homo sapiens (Human)	860,1944	1718,3742	1717,8747	0,4995	30,84	0	NLDIERPTYTNLNR		1
TBA8_HUMAN	Tubulin alpha-8 chain - Homo sapiens (Human)	938,3579	1874,7013	1873,9758	0,7255	26,79	1	RNLDIERPTYTNLNR		2
TBA8_HUMAN	Tubulin alpha-8 chain - Homo sapiens (Human)	1826,2119	1825,2046	1823,9782	1,2265	37,22	0	VGINYQPPTVVPGGDLAK		1
TBB1_HUMAN	Tubulin beta-1 chain - Homo sapiens (Human)	566,1139	1130,2133	1129,588	0,6253	58,15	0	FPGQLNADLR		4
TBB1_HUMAN	Tubulin beta-1 chain - Homo sapiens (Human)	629,9329	1257,8512	1257,683	0,1682	44,54	1	FPGQLNADLRK		1
TBB1_HUMAN	Tubulin beta-1 chain - Homo sapiens (Human)	539,6005	1077,1864	1076,525	0,6613	40,38	1	IREEYPDR		2
TBB1_HUMAN	Tubulin beta-1 chain - Homo sapiens (Human)	572,5064	1142,9983	1142,627	0,3712	63,33	0	LAVNMVPFPR		1
TBB1_HUMAN	Tubulin beta-1 chain - Homo sapiens (Human)	580,4682	1158,9218	1158,6219	0,2998	50,67	0	LAVNMVPFPR	Oxidation (M)	1
TBB1_HUMAN	Tubulin beta-1 chain - Homo sapiens (Human)	704,3878	2110,1417	2110,0775	0,0642	16,85	1	MREIVHIQIGQCGNQIGAK	Oxidation (M)	1
TBB1_HUMAN	Tubulin beta-1 chain - Homo sapiens (Human)	834,7886	2501,3441	2502,2406	-0,8965	4,03	3	NTMAACDLRRGRYLTVACIFR	Carbamidomethyl (C); Oxidation (M)	1
TBB2A_HUMAN	Tubulin beta-2A chain - Homo sapiens (Human)	808,6266	1615,2387	1614,8287	0,41	31,24	0	AILVDLEPGTMDSVR		1
TBB2A_HUMAN	Tubulin beta-2A chain - Homo sapiens (Human)	544,3745	1630,1018	1630,8236	-0,7218	9,1	0	AILVDLEPGTMDSVR	Oxidation (M)	1
TBB2A_HUMAN	Tubulin beta-2A chain - Homo sapiens (Human)	912,2434	1822,4722	1821,9156	0,5566	68,26	0	EIVHIQAGQCGNQIGAK	Carbamidomethyl (C)	2
TBB2A_HUMAN	Tubulin beta-2A chain - Homo sapiens (Human)	1446,6107	1445,6034	1445,682	-0,0786	47,05	0	EVDEQMLNVQNK		2
TBB2A_HUMAN	Tubulin beta-2A chain - Homo sapiens (Human)	732,0783	1462,142	1461,6769	0,465	55,61	0	EVDEQMLNVQNK	Oxidation (M)	1
TBB2A_HUMAN	Tubulin beta-2A chain - Homo sapiens (Human)	566,0393	1130,064	1129,588	0,476	48,43	0	FPGQLNADLR		1
TBB2A_HUMAN	Tubulin beta-2A chain - Homo sapiens (Human)	629,9329	1257,8512	1257,683	0,1682	44,54	1	FPGQLNADLRK		1
TBB2A_HUMAN	Tubulin beta-2A chain - Homo sapiens (Human)	980,5097	1959,0048	1957,9745	1,0302	75,32	0	GHYTEGAELVDSVLDVVR		1
TBB2A_HUMAN	Tubulin beta-2A chain - Homo sapiens (Human)	724,0955	2169,2646	2168,0024	1,2622	34,76	2	GRMSMKEVDEQMLNVQNK	2 Oxidation (M)	1
TBB2A_HUMAN	Tubulin beta-2A chain - Homo sapiens (Human)	539,6005	1077,1864	1076,525	0,6613	40,38	1	IREEYPDR		2
TBB2A_HUMAN	Tubulin beta-2A chain - Homo sapiens (Human)	615,3695	1228,7245	1228,591	0,1334	64,24	0	ISEQFTAMFR		1
TBB2A_HUMAN	Tubulin beta-2A chain - Homo sapiens (Human)	623,729	1245,4434	1244,586	0,8574	53,96	0	ISEQFTAMFR	Oxidation (M)	1
TBB2A_HUMAN	Tubulin beta-2A chain - Homo sapiens (Human)	572,5064	1142,9983	1142,627	0,3712	63,33	0	LAVNMVPFPR		1
TBB2A_HUMAN	Tubulin beta-2A chain - Homo sapiens (Human)	580,4682	1158,9218	1158,6219	0,2998	50,67	0	LAVNMVPFPR	Oxidation (M)	1
TBB2A_HUMAN	Tubulin beta-2A chain - Homo sapiens (Human)	903,7861	2708,3365	2707,331	1,0056	45,22	0	LTTPTYGDLNHLVSATMSGVTTCLR	Carbamidomethyl (C)	1
TBB2A_HUMAN	Tubulin beta-2A chain - Homo sapiens (Human)	704,3878	2110,1417	2109,0571	1,0846	79,42	1	MREIVHIQAGQCGNQIGAK	Carbamidomethyl (C)	1
TBB2A_HUMAN	Tubulin beta-2A chain - Homo sapiens (Human)	709,6064	2125,7974	2125,0521	0,7454	58,5	1	MREIVHIQAGQCGNQIGAK	Carbamidomethyl (C); Oxidation (M)	1
TBB2A_HUMAN	Tubulin beta-2A chain - Homo sapiens (Human)	929,9986	1857,9826	1856,9342	1,0484	87,35	0	MSATFIGNSTAIQELFK		1
TBB2A_HUMAN	Tubulin beta-2A chain - Homo sapiens (Human)	849,2254	1696,4362	1695,8257	0,6105	46,36	0	NSSYFVEWIPNNVK		3
TBB2A_HUMAN	Tubulin beta-2A chain - Homo sapiens (Human)	1400,4138	2798,8131	2797,3361	1,477	55,78	0	SGPFGQIFRPDNFVFGQSGAGNNWAK		1
TBB2A_HUMAN	Tubulin beta-2A chain - Homo sapiens (Human)	514,8352	1027,6558	1027,5121	0,1437	62,84	0	TAVCDIPPR	Carbamidomethyl (C)	1
TBB2C_HUMAN	Tubulin beta-2C chain - Homo sapiens (Human)	846,7248	1691,435	1690,86	0,575	71,33	0	ALTVPELTQQMFDAK		1
								AVLVDLEPGTMDSVR		

TBB2C_HUMAN	Tubulin beta-2C chain - Homo sapiens (Human)	809,8948	1617,775	1616,808	0,967	56,27	0	AVLVDLEPGTMDSVR	Oxidation (M)	1
TBB2C_HUMAN	Tubulin beta-2C chain - Homo sapiens (Human)	912,2434	1822,4722	1821,9156	0,5566	68,26	0	EIVHLQAGQCGNQIGAK	Carbamidomethyl (C)	2
TBB2C_HUMAN	Tubulin beta-2C chain - Homo sapiens (Human)	1446,6107	1445,6034	1445,682	-0,0786	47,05	0	EVDEQMLNVQNK		2
TBB2C_HUMAN	Tubulin beta-2C chain - Homo sapiens (Human)	732,0783	1462,142	1461,6769	0,465	55,61	0	EVDEQMLNVQNK	Oxidation (M)	1
TBB2C_HUMAN	Tubulin beta-2C chain - Homo sapiens (Human)	566,0393	1130,064	1129,588	0,476	48,43	0	FPGQLNADLR		1
TBB2C_HUMAN	Tubulin beta-2C chain - Homo sapiens (Human)	629,9329	1257,8512	1257,683	0,1682	44,54	1	FPGQLNADLRK		1
TBB2C_HUMAN	Tubulin beta-2C chain - Homo sapiens (Human)	980,5097	1959,0048	1957,9745	1,0302	75,32	0	GHYTEGAELVDSVLDVVR		1
TBB2C_HUMAN	Tubulin beta-2C chain - Homo sapiens (Human)	724,0955	2169,2646	2168,0024	1,2622	34,76	2	GRMSMKEVDEQMLNVQNK	2 Oxidation (M)	1
TBB2C_HUMAN	Tubulin beta-2C chain - Homo sapiens (Human)	660,596	1319,1775	1318,6955	0,482	57,23	0	IMNTFSVVPSPK		1
TBB2C_HUMAN	Tubulin beta-2C chain - Homo sapiens (Human)	668,775	1335,5355	1334,6904	0,845	61,85	0	IMNTFSVVPSPK	Oxidation (M)	1
TBB2C_HUMAN	Tubulin beta-2C chain - Homo sapiens (Human)	1328,5186	1327,5113	1327,6408	-0,1295	60,02	0	INVYYNEATGGK		2
TBB2C_HUMAN	Tubulin beta-2C chain - Homo sapiens (Human)	539,6005	1077,1864	1076,525	0,6613	40,38	1	IREEYPDR		2
TBB2C_HUMAN	Tubulin beta-2C chain - Homo sapiens (Human)	615,3695	1228,7245	1228,591	0,1334	64,24	0	ISEQFTAMFR		1
TBB2C_HUMAN	Tubulin beta-2C chain - Homo sapiens (Human)	623,729	1245,4434	1244,586	0,8574	53,96	0	ISEQFTAMFR	Oxidation (M)	1
TBB2C_HUMAN	Tubulin beta-2C chain - Homo sapiens (Human)	572,5064	1142,9983	1142,627	0,3712	63,33	0	LAVNMVPFPR		1
TBB2C_HUMAN	Tubulin beta-2C chain - Homo sapiens (Human)	580,4682	1158,9218	1158,6219	0,2998	50,67	0	LAVNMVPFPR	Oxidation (M)	1
TBB2C_HUMAN	Tubulin beta-2C chain - Homo sapiens (Human)	903,7861	2708,3365	2707,331	1,0056	45,22	0	LTTPTYGDLNHLVSATMSGVTTCLR	Carbamidomethyl (C)	1
TBB2C_HUMAN	Tubulin beta-2C chain - Homo sapiens (Human)	704,3878	2110,1417	2109,0571	1,0846	79,42	1	MREIVHLQAGQCGNQIGAK	Carbamidomethyl (C)	1
TBB2C_HUMAN	Tubulin beta-2C chain - Homo sapiens (Human)	709,6064	2125,7974	2125,0521	0,7454	58,5	1	MREIVHLQAGQCGNQIGAK	Carbamidomethyl (C); Oxidation (M)	1
TBB2C_HUMAN	Tubulin beta-2C chain - Homo sapiens (Human)	929,9986	1857,9826	1856,9342	1,0484	87,35	0	MSATFIGNSTAIQELFK		1
TBB2C_HUMAN	Tubulin beta-2C chain - Homo sapiens (Human)	849,2254	1696,4362	1695,8257	0,6105	46,36	0	NSSYFVEWIPNNVK		3
TBB2C_HUMAN	Tubulin beta-2C chain - Homo sapiens (Human)	1400,4138	2798,8131	2797,3361	1,477	55,78	0	SGPFGQIFRPDNFVFGQSGAGNNWAK		1
TBB2C_HUMAN	Tubulin beta-2C chain - Homo sapiens (Human)	514,8352	1027,6558	1027,5121	0,1437	62,84	0	TAVCDIPPR	Carbamidomethyl (C)	1
TBB3_HUMAN	Tubulin beta-3 chain - Homo sapiens (Human)	808,6266	1615,2387	1614,8287	0,41	31,24	0	AILVDLEPGTMDSVR		1
TBB3_HUMAN	Tubulin beta-3 chain - Homo sapiens (Human)	544,3745	1630,1018	1630,8236	-0,7218	9,1	0	AILVDLEPGTMDSVR	Oxidation (M)	1
TBB3_HUMAN	Tubulin beta-3 chain - Homo sapiens (Human)	846,7248	1691,435	1690,86	0,575	71,33	0	ALTVPELTQQMFDAK		1
TBB3_HUMAN	Tubulin beta-3 chain - Homo sapiens (Human)	912,2434	1822,4722	1821,9156	0,5566	68,26	0	EIVHIQAGQCGNQIGAK	Carbamidomethyl (C)	2
TBB3_HUMAN	Tubulin beta-3 chain - Homo sapiens (Human)	696,0993	1390,1841	1389,681	0,5031	4,22	0	EVDEQMLAIQSK		1
TBB3_HUMAN	Tubulin beta-3 chain - Homo sapiens (Human)	566,1769	1130,3392	1129,588	0,7512	49,31	0	FPGQLNADLR		2
TBB3_HUMAN	Tubulin beta-3 chain - Homo sapiens (Human)	629,9329	1257,8512	1257,683	0,1682	44,54	1	FPGQLNADLRK		1
TBB3_HUMAN	Tubulin beta-3 chain - Homo sapiens (Human)	980,5097	1959,0048	1957,9745	1,0302	75,32	0	GHYTEGAELVDSVLDVVR		1
TBB3_HUMAN	Tubulin beta-3 chain - Homo sapiens (Human)	668,775	1335,5355	1334,6904	0,845	61,85	0	IMNTFSVVPSPK	Oxidation (M)	1
TBB3_HUMAN	Tubulin beta-3 chain - Homo sapiens (Human)	660,4658	1318,917	1318,6955	0,2215	75,98	0	IMNTFSVVPSPK		2
TBB3_HUMAN	Tubulin beta-3 chain - Homo sapiens (Human)	615,3695	1228,7245	1228,591	0,1334	64,24	0	ISEQFTAMFR		1
TBB3_HUMAN	Tubulin beta-3 chain - Homo sapiens (Human)	623,5592	1245,1038	1244,586	0,5178	70,84	0	ISEQFTAMFR	Oxidation (M)	2
TBB3_HUMAN	Tubulin beta-3 chain - Homo sapiens (Human)	572,5064	1142,9983	1142,627	0,3712	63,33	0	LAVNMVPFPR		1
TBB3_HUMAN	Tubulin beta-3 chain - Homo sapiens (Human)	580,4682	1158,9218	1158,6219	0,2998	50,67	0	LAVNMVPFPR	Oxidation (M)	1
TBB3_HUMAN	Tubulin beta-3 chain - Homo sapiens (Human)	704,3878	2110,1417	2109,0571	1,0846	79,42	1	MREIVHIQAGQCGNQIGAK	Carbamidomethyl (C)	1
TBB3_HUMAN	Tubulin beta-3 chain - Homo sapiens (Human)	709,6064	2125,7974	2125,0521	0,7454	58,5	1	MREIVHIQAGQCGNQIGAK	Carbamidomethyl (C); Oxidation (M)	1
TBB3_HUMAN	Tubulin beta-3 chain - Homo sapiens (Human)	849,2254	1696,4362	1695,8257	0,6105	46,36	0	NSSYFVEWIPNNVK		3
TBB4_HUMAN	Tubulin beta-4 chain - Homo sapiens (Human)	846,7248	1691,435	1690,86	0,575	71,33	0	ALTVPELTQQMFDAK		1
TBB4_HUMAN	Tubulin beta-4 chain - Homo sapiens (Human)	801,506	1600,9975	1600,8131	0,1844	50,08	0	AVLVDLEPGTMDSVR		1
TBB4_HUMAN	Tubulin beta-4 chain - Homo sapiens (Human)	809,8948	1617,775	1616,808	0,967	56,27	0	AVLVDLEPGTMDSVR	Oxidation (M)	1
TBB4_HUMAN	Tubulin beta-4 chain - Homo sapiens (Human)	912,2434	1822,4722	1821,9156	0,5566	68,26	0	EIVHLQAGQCGNQIGAK	Carbamidomethyl (C)	2
TBB4_HUMAN	Tubulin beta-4 chain - Homo sapiens (Human)	566,0393	1130,064	1129,588	0,476	48,43	0	FPGQLNADLR		1
TBB4_HUMAN	Tubulin beta-4 chain - Homo sapiens (Human)	629,9329	1257,8512	1257,683	0,1682	44,54	1	FPGQLNADLRK		1
TBB4_HUMAN	Tubulin beta-4 chain - Homo sapiens (Human)	971,7497	1941,4848	1941,9796	-0,4948	0,84	0	GHYTEGAELVDAVLDVVR		1
TBB4_HUMAN	Tubulin beta-4 chain - Homo sapiens (Human)	660,596	1319,1775	1318,6955	0,482	57,23	0	IMNTFSVVPSPK		1
TBB4_HUMAN	Tubulin beta-4 chain - Homo sapiens (Human)	668,775	1335,5355	1334,6904	0,845	61,85	0	IMNTFSVVPSPK	Oxidation (M)	1
TBB4_HUMAN	Tubulin beta-4 chain - Homo sapiens (Human)	615,3695	1228,7245	1228,591	0,1334	64,24	0	ISEQFTAMFR		1
TBB4_HUMAN	Tubulin beta-4 chain - Homo sapiens (Human)	623,729	1245,4434	1244,586	0,8574	53,96	0	ISEQFTAMFR	Oxidation (M)	1
TBB4_HUMAN	Tubulin beta-4 chain - Homo sapiens (Human)	572,5064	1142,9983	1142,627	0,3712	63,33	0	LAVNMVPFPR		1
TBB4_HUMAN	Tubulin beta-4 chain - Homo sapiens (Human)	580,4682	1158,9218	1158,6219	0,2998	50,67	0	LAVNMVPFPR	Oxidation (M)	1
TBB4_HUMAN	Tubulin beta-4 chain - Homo sapiens (Human)	903,7861	2708,3365	2707,331	1,0056	45,22	0	LTTPTYGDLNHLVSATMSGVTTCLR	Carbamidomethyl (C)	1
TBB4_HUMAN	Tubulin beta-4 chain - Homo sapiens (Human)	929,9986	1857,9826	1856,9342	1,0484	88,69	0	MAATFIGNSTAIQELFK	Oxidation (M)	1
TBB4_HUMAN	Tubulin beta-4 chain - Homo sapiens (Human)	704,3878	2110,1417	2109,0571	1,0846	79,42	1	MREIVHLQAGQCGNQIGAK	Carbamidomethyl (C)	1
								MREIVHLQAGQCGNQIGAK		

TBB4_HUMAN	Tubulin beta-4 chain - Homo sapiens (Human)	849,2254	1696,4362	1695,8257	0,6105	46,36	0	NSSYFVEWIPNNVK		3
TBB4_HUMAN	Tubulin beta-4 chain - Homo sapiens (Human)	1400,4138	2798,8131	2797,3361	1,477	55,78	0	SGPFGQIFRPDNFVFGQSGAGNNWAK		1
TBB4_HUMAN	Tubulin beta-4 chain - Homo sapiens (Human)	514,8352	1027,6558	1027,5121	0,1437	62,84	0	TAVCDIPPR	Carbamidomethyl (C)	1
TBB5_HUMAN	Tubulin beta chain - Homo sapiens (Human)	808,6266	1615,2387	1614,8287	0,41	31,24	0	AILVDLEPGTMDSVR		1
TBB5_HUMAN	Tubulin beta chain - Homo sapiens (Human)	544,3745	1630,1018	1630,8236	-0,7218	9,1	0	AILVDLEPGTMDSVR	Oxidation (M)	1
TBB5_HUMAN	Tubulin beta chain - Homo sapiens (Human)	830,8528	1659,6911	1658,8879	0,8031	57,02	0	ALTVPELTQQVFDAK		1
TBB5_HUMAN	Tubulin beta chain - Homo sapiens (Human)	912,2434	1822,4722	1821,9156	0,5566	68,26	0	EIVHIQAGQCGNQIGAK	Carbamidomethyl (C)	2
TBB5_HUMAN	Tubulin beta chain - Homo sapiens (Human)	732,0783	1462,142	1461,6769	0,465	55,61	0	EVDEQMLNVQNK	Oxidation (M)	1
TBB5_HUMAN	Tubulin beta chain - Homo sapiens (Human)	724,0189	1446,0233	1445,682	0,3413	70,4	0	EVDEQMLNVQNK		4
TBB5_HUMAN	Tubulin beta chain - Homo sapiens (Human)	566,1769	1130,3392	1129,588	0,7512	49,31	0	FPGQLNADLR		3
TBB5_HUMAN	Tubulin beta chain - Homo sapiens (Human)	629,9329	1257,8512	1257,683	0,1682	44,54	1	FPGQLNADLRK		1
TBB5_HUMAN	Tubulin beta chain - Homo sapiens (Human)	980,5097	1959,0048	1957,9745	1,0302	75,32	0	GHYTEGAELVDSVLDVVR		1
TBB5_HUMAN	Tubulin beta chain - Homo sapiens (Human)	724,0202	2169,0388	2168,0024	1,0364	58,42	2	GRMSMKEVDEQMLNVQNK	2 Oxidation (M)	3
TBB5_HUMAN	Tubulin beta chain - Homo sapiens (Human)	668,775	1335,5355	1334,6904	0,845	61,85	0	IMNTFSVVPSPK	Oxidation (M)	1
TBB5_HUMAN	Tubulin beta chain - Homo sapiens (Human)	660,4658	1318,917	1318,6955	0,2215	75,98	0	IMNTFSVVPSPK		2
TBB5_HUMAN	Tubulin beta chain - Homo sapiens (Human)	539,6005	1077,1864	1076,525	0,6613	40,38	1	IREEYPDR		2
TBB5_HUMAN	Tubulin beta chain - Homo sapiens (Human)	615,3695	1228,7245	1228,591	0,1334	64,24	0	ISEQFTAMFR		1
TBB5_HUMAN	Tubulin beta chain - Homo sapiens (Human)	623,5592	1245,1038	1244,586	0,5178	70,84	0	ISEQFTAMFR	Oxidation (M)	2
TBB5_HUMAN	Tubulin beta chain - Homo sapiens (Human)	651,4918	1300,9691	1300,6299	0,3392	69,06	0	ISVYYNEATGGK		4
TBB5_HUMAN	Tubulin beta chain - Homo sapiens (Human)	572,5064	1142,9983	1142,627	0,3712	63,33	0	LAVNMVPFPR		1
TBB5_HUMAN	Tubulin beta chain - Homo sapiens (Human)	580,4682	1158,9218	1158,6219	0,2998	50,67	0	LAVNMVPFPR	Oxidation (M)	1
TBB5_HUMAN	Tubulin beta chain - Homo sapiens (Human)	903,7861	2708,3365	2707,331	1,0056	45,22	0	LTTPTYGDLNHLVSATMSGVTTCLR	Carbamidomethyl (C)	1
TBB5_HUMAN	Tubulin beta chain - Homo sapiens (Human)	935,8248	1869,6351	1868,9706	0,6645	79,91	0	MAVTFIGNSTAIQELFK		1
TBB5_HUMAN	Tubulin beta chain - Homo sapiens (Human)	944,0869	1886,1593	1884,9655	1,1937	93,34	0	MAVTFIGNSTAIQELFK	Oxidation (M)	2
TBB5_HUMAN	Tubulin beta chain - Homo sapiens (Human)	704,3878	2110,1417	2109,0571	1,0846	79,42	1	MREIVHIQAGQCGNQIGAK	Carbamidomethyl (C)	1
TBB5_HUMAN	Tubulin beta chain - Homo sapiens (Human)	709,6064	2125,7974	2125,0521	0,7454	58,5	1	MREIVHIQAGQCGNQIGAK	Carbamidomethyl (C); Oxidation (M)	1
TBB5_HUMAN	Tubulin beta chain - Homo sapiens (Human)	849,2254	1696,4362	1695,8257	0,6105	46,36	0	NSSYFVEWIPNNVK		3
TBB5_HUMAN	Tubulin beta chain - Homo sapiens (Human)	1400,4138	2798,8131	2797,3361	1,477	55,78	0	SGPFGQIFRPDNFVFGQSGAGNNWAK		1
TBB5_HUMAN	Tubulin beta chain - Homo sapiens (Human)	514,8352	1027,6558	1027,5121	0,1437	62,84	0	TAVCDIPPR	Carbamidomethyl (C)	1
TBB6_HUMAN	Tubulin beta-6 chain - Homo sapiens (Human)	566,0393	1130,064	1129,588	0,476	48,43	0	FPGQLNADLR		1
TBB6_HUMAN	Tubulin beta-6 chain - Homo sapiens (Human)	629,9329	1257,8512	1257,683	0,1682	44,54	1	FPGQLNADLRK		1
TBB6_HUMAN	Tubulin beta-6 chain - Homo sapiens (Human)	971,7497	1941,4848	1941,9796	-0,4948	0,84	0	GHYTEGAELVDAVLDVVR		1
TBB6_HUMAN	Tubulin beta-6 chain - Homo sapiens (Human)	572,5064	1142,9983	1142,627	0,3712	63,33	0	LAVNMVPFPR		1
TBB6_HUMAN	Tubulin beta-6 chain - Homo sapiens (Human)	580,4682	1158,9218	1158,6219	0,2998	50,67	0	LAVNMVPFPR	Oxidation (M)	1
TBB6_HUMAN	Tubulin beta-6 chain - Homo sapiens (Human)	929,9986	1857,9826	1856,9342	1,0484	87,35	0	MASTFIGNSTAIQELFK		1
TBB6_HUMAN	Tubulin beta-6 chain - Homo sapiens (Human)	849,2254	1696,4362	1695,8257	0,6105	46,36	0	NSSYFVEWIPNNVK		3
TBB8_HUMAN	Tubulin beta-8 chain - Homo sapiens (Human)	566,1769	1130,3392	1129,588	0,7512	49,31	0	FPGQLNADLR		1
TBB8_HUMAN	Tubulin beta-8 chain - Homo sapiens (Human)	943,8093	1885,6041	1883,9451	1,659	5,79	0	MSATFIGNNTAIQELFK		1
TNKS1_HUMAN	Tankyrase-1	626,1507	1875,4303	1875,0036	0,4267	8,86	0	IDVCIVLLQHGADPNIR		1
TNKS1_HUMAN	Tankyrase-1	508,5311	1015,0476	1013,5505	1,497	31,02	0	LVDAANVNAK		1
WBP4_HUMAN	WW domain-binding protein 4 - Homo sapiens (Human)	626,5385	1251,0624	1249,6455	1,4169	10,13	2	ADYWKSQPKK		1
								NSDGGSDPETQKEK		
